# Monthly Trends in the Life Events Reported in the Prior Year and First Year of the COVID-19 Pandemic in New Zealand

**DOI:** 10.3389/fpsyg.2022.829643

**Published:** 2022-03-11

**Authors:** Chloe Howard, Nickola C. Overall, Chris G. Sibley

**Affiliations:** School of Psychology, University of Auckland, Auckland, New Zealand

**Keywords:** life events, stressors, COVID-19 pandemic, gender, longitudinal analysis

## Abstract

The current study examines changes in the economic, social, and well-being life events that women and men reported during the first 7 months of the COVID-19 pandemic. Analyses compared monthly averages in cross-sectional national probability data from two annual waves of the New Zealand Attitudes and Values Study collected between October 2018–September 2019 (*N* = 17,924), and October 2019–September 2020 (*N* = 41,653), which included the first 7 months of the pandemic (Mar–Sep 2020). Results indicated that people (particularly women) reported increased job loss in the months following an initial COVID-19 lockdown relative to the same months the year earlier. Women also experienced an increase in family troubles when restrictions eased and reported increased negative lifestyle changes that persisted throughout the first 7 months of the pandemic. The proportion of people experiencing many other life events (e.g., mental health, financial concerns) in New Zealand did not differ reliably from the pre-pandemic monthly baseline. These results highlight resilience to many potential negative life events within the first 7 months of the COVID-19 pandemic. However, the pandemic did not affect everyone equally, and the burden of increased negative events appears more heavily borne by women. As the pandemic continues more than 18 months from initial community transmission of COVID-19, our findings provide important insight into the impact of the pandemic on potential negative life events, especially among women, that may have critical consequences for mental health, gender equality, and social well-being over time.

## Introduction

The COVID-19 pandemic has impacted all aspects of daily living (Gruber et al., 2020). In New Zealand, a series of national lockdowns contained the emergence of COVID-19 in the community during the first year of the pandemic (New Zealand Government, 2020). The initial mandatory lockdown required people to stay home except for essential movement, creating widespread challenges to economic, social, and personal well-being. Published data reflects these challenges by revealing that many people have faced a range of economic, social, and well-being life events during the pandemic (e.g., Jean-Baptiste et al., 2020). However, extant research currently lacks both prospective data from before the pandemic as well as data beyond the initial few months of the pandemic. In the current research, we expand upon cross-sectional studies conducted within a short time period at the start of the pandemic (e.g., 7–9 April 2020; e.g., Park et al., 2020) to examine pre- versus post-pandemic differences in reported economic, social, and well-being life events. We draw on data from the New Zealand Attitudes and Values Study (NZAVS) collected the year before (2018/2019; *N* = 17,924), and during (2019/2020; *N* = 41,653), the pandemic to examine whether the monthly proportions of women and men reporting economic, social, and well-being life events changed during the first 7 months of the pandemic relative to the same trend 12 months earlier.

Research has predominantly focused on documenting the impact of the pandemic on people’s mental health (for an overview, see Clemente-Suárez et al. (2020)). Globally, studies show that the pandemic and associated lockdowns were associated with a range of negative mental health outcomes, including greater psychological distress, poorer sleep, higher levels of perceived stress, and greater depressive, anxiety, and post-traumatic stress symptoms (Alkhamees et al., 2020; Rossi et al., 2020, 2021c; Goularte et al., 2021). In terms of risk factors, one study conducted by Chandola et al. (2020) provides initial evidence that the occurrence of life events during the pandemic, including unemployment, financial difficulties, and loneliness, increased the likelihood of greater psychological distress. This initial finding highlights the importance of assessing the impact of the pandemic on changes in a range of life events to further understand the breadth of repercussions the COVID-19 pandemic likely has had on people’s lives, including mental health, gender equality, job security, and social well-being. Thus, in the current research we examine a range of positive and negative life events across economic, social, and well-being domains that people may have experienced during the pandemic.

Published data pinpoints pandemic, economic, social, and well-being events as the most prevalent COVID-19 stressors (e.g., Jean-Baptiste et al., 2020; Park et al., 2020). Pandemic stressors frequently reported include news exposure about the severity and contagiousness of COVID-19 and uncertainty over the duration of lockdowns and social restrictions (McGinty et al., 2020; Park et al., 2020; Moore et al., 2021; Whitehead and Torossian, 2021). Economic stressors commonly reported include job loss, financial concerns, work-related challenges (e.g., working from home), and job insecurity (Jean-Baptiste et al., 2020; Kujawa et al., 2020; McGinty et al., 2020; Park et al., 2020). Social events commonly reported include greater racism, concern for loved ones, increased social support (Jean-Baptiste et al., 2020), and changes in time spent with family and friends (Kujawa et al., 2020; Park et al., 2020; Moore et al., 2021; Whitehead and Torossian, 2021). Lastly, well-being events frequently reported include poorer mental health, isolation, loneliness, and fear of the unknown (Jean-Baptiste et al., 2020; Entringer and Gosling, 2021; Moore et al., 2021; Whitehead and Torossian, 2021), along with negative changes to lifestyle and personal care routines, such as housework and socializing (e.g., Constant et al., 2020; Park et al., 2020; Giuntella et al., 2021).

Yet, other work suggests that resilience is a common response to adversity, including disease outbreak (see Chen and Bonanno (2020)), and thus these experiences may not be perceived as significant or stressful life events. Resilience is defined as the maintenance or stability of healthy psychological functioning during adversity (Bonanno, 2004) and measured in several ways, including the absence of distress along with people’s access to a range of individual (e.g., coping strategies) and environmental resources (e.g., social support; Connor and Davidson, 2003; Windle et al., 2008; Rossi et al., 2021b). Resilience is most often studied in relation to adverse life events, with research indicating that large proportion of people show resilience following stressful life events, including trauma and bereavement (Bonanno, 2004). However, research on prior pandemics (e.g., SARS) suggest that severe stressors, such as disease outbreak, result in lower levels of resilience, although this research primarily relies on cross-sectional data (Chen and Bonanno, 2020). By contrast, initial longitudinal research provides early evidence of resilience during the COVID-19 pandemic. Based on their review of studies examining well-being during the first year of the pandemic, Aknin et al. (2021) concluded that psychological distress increased in the first months of the pandemic, but returned to baseline levels by mid-2020, and average levels of other well-being variables (e.g., life satisfaction) were relatively unaffected throughout the pandemic. Average resilience in New Zealand was also evident in Sibley et al. (2020) examination of the effects of COVID-19 national lockdown. Their analysis comparing participants who completed the NZAVS survey in the first 18 days of lockdown (post-lockdown) to propensity score matched participants who completed the survey before the pandemic (pre-lockdown) revealed only slight increases in mental distress and no differences in rumination, life satisfaction, and perceived social support (also Davis et al. (2021)). Sibley et al. (2020) also found that the post-lockdown group reported less fatigue, and greater national identification as well as trust in science, politicians, and police, than the pre-lockdown group. Other positive changes have also been noted in New Zealand, including improved interpersonal relationships, reduced financial insecurity from government-provided wage subsidies, and better self-care and personal development (Jenkins et al., 2021).

Given that life events play a powerful role in determining life satisfaction (see Luhmann et al. (2012)), depression (see Hammen (2005)), and physical illness (see Cohen et al. (2019)), the presence of significant life events along with potential resilience in the face of pandemic challenges offers a mixed picture of the effects of the pandemic. Examinations of people’s reported life events indicate that people are experiencing a range of stressful life events, whereas examination of broad indicators of psychological and social well-being suggest there may be some relative resilience. However, most studies examining life events referenced above employed retrospective and concurrent research designs (e.g., Jean-Baptiste et al., 2020; Park et al., 2020; Moore et al., 2021), meaning there is a lack of data on stressors prior to the pandemic to examine whether the pandemic changed reports of life events. Moreover, studies that have employed longitudinal designs to assess changes in well-being have examined effects at the start of the pandemic (e.g., Sibley et al., 2020) limiting understanding of the ongoing effects of the pandemic on people’s lives. By examining pre- versus post-pandemic changes in life events across the first 7 months of the pandemic, the current research should help identify relative risk versus resilience in economic, social, and well-being domains. The results will be informative to the development of policy and resource distribution to mitigate potential negative impacts of the ongoing challenges associated with the pandemic (Gruber et al., 2020).

## Materials and Methods

### Overview of the Current Study

**FIGURE 1:**
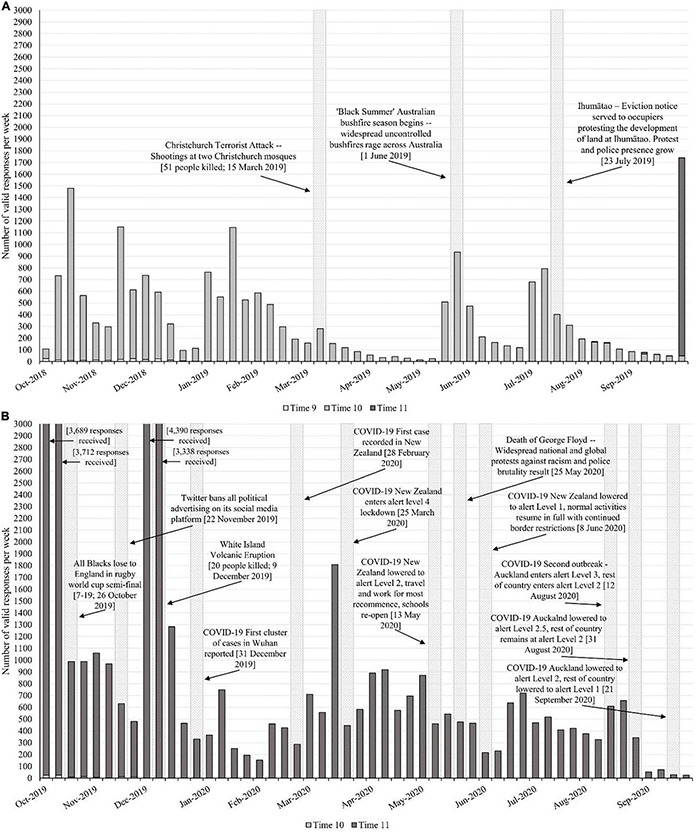
Timelines of monthly valid responses from the NZAVS in Time 10 **(A)** and Time 11 **(B)**, compared with earlier waves and in the context of significant events (e.g., COVID-19, Christchurch Terrorist Attack).

We analyze monthly data collected prior to and during the pandemic to examine pre- versus post-pandemic reports of economic, social, and well-being life events that people experienced. We leverage data from the NZAVS—a nationwide longitudinal panel study that since 2009 has gathered annual assessments of various psychological variables based on a random sample of the electoral roll. The NZAVS is an omnibus survey that measures a wide variety of variables through Likert scales (e.g., personality) and open-ended responses that are coded (e.g., gender, life events) from either established psychological scales or measures designed specifically for the NZAVS. Participants complete either an online or physical copy of the survey each year. Data collection is staggered throughout the year to ensure participants are contacted approximately 1 year after they completed the last survey (for more information on the NZAVS, please visit the website^1^ or the NZAVS supporting information OSF^2^). Figure 1 shows the number of valid responses per week across the two annual waves of data collection before (2018/2019) and during (2019/2020) the pandemic. Hundreds of respondents completed the survey each month (Oct–Sep) in the annual wave prior to the pandemic (2018/2019; average *N* per month = 1,520), and the wave during the pandemic (2019/2020; average *N* per month = 3,551). Thus, the NZAVS provides data for thousands of participants (*N* = 59,577) that completed the survey before and during the pandemic (Mar–Sep 2020; *N* = 15,728) allowing us to test whether the pandemic increased rates of life events that diverged from monthly trends observed the preceding year.

Extending existing research using checklists developed to measure specific COVID-19 stressors (e.g., Park et al., 2020), we use the Broad Inventory of Specific Life Events (BISLE; Howard et al., 2022), which uniquely assesses a broad range of life events that people may experience each year. The BISLE captures events reported as occurring any time over the past year through a combination of select probe events and open-ended descriptions of events generated by participants, which are then coded. Reported life events in a given month thus reflect events that have occurred in that month as well as events over the past year that were particularly salient to participants when completing the survey. Because the BISLE asks participants if any important life changes occurred in the past year without prompt of the pandemic, we examine differential proportions in reported life events in the context of COVID-19, rather than priming participants to report specific COVID-19 stressors (e.g., Jean-Baptiste et al., 2020; Park et al., 2020). This provides valid comparisons of events reported pre- and post-pandemic. For example, a pattern of high rates of reported pandemic-related events, such as virus concerns, lockdowns, and vaccines, from March 2020 onward compared to no reported pandemic-related events in 2018/2019 would illustrate that people are spontaneously reporting relevant events in their lives.

We compare monthly trends from the first 7 months of the pandemic in New Zealand (Mar–Sep 2020) to the monthly trend observed in the year prior to the pandemic (e.g., comparing May 2020 to May 2019). This approach means that any observed differences in events from March 2020 onward compared to the same months in the prior year are due to pandemic-related conditions rather than seasonal periodicity that might have coincided with the onset of the pandemic. This approach also allows us to demonstrate the specific pattern of differences, including whether any changes are persistent across the months of the pandemic or increase at certain points. For example, job loss may increase from the onset of the pandemic (March 2020) and continually increase compared to the prior year as the pandemic reduced available resources for businesses to continue employment activity over time. Although at the time of writing it is more than 18 months since the start of the pandemic, our study examining the first 7 months of the pandemic covers unprecedented societal shifts that altered how people conducted their lives, such as national lockdowns and strict legally enforced and voluntary restrictions. This allows us to provide important insight into how the pandemic impacted reports of life events that can inform future policy and research as the pandemic continues over upcoming months and years.

We conduct these assessments separately for women and men because (1) prior research has established systematic differences in the reporting of events between women and men (see Davis et al. (1999) and Dohrenwend (2006)), and (2) there is evidence that the challenges of the COVID-19 pandemic disproportionally affect women (Azcona et al., 2020; Stats NZ, 2020a; UN Women, 2020). Job loss and insecurity are higher among women in New Zealand and globally due to women often occupying jobs in industries most affected by the pandemic (e.g., hospitality; Azcona et al., 2020; Stats NZ, 2020a; UN Women, 2020). Lockdowns also meant that women took on more childcare responsibilities and unpaid labor (Waddell et al., 2021), and placed women at a higher risk of loneliness and social isolation (Bu et al., 2020; Entringer and Gosling, 2021). Lockdowns have also been accompanied by increases in domestic violence against women (Azcona et al., 2020; UN Women, 2020).

### Sampling Procedure, Participants, and Retention Rates

We draw data from (1) Time 10 of the NZAVS, which occurred between October 2018 and September 2019 (47,951 participants) and (2) Time 11, which occurred between October 2019 and September 2020 (42,684 participants) and covered the first 7 months of the pandemic (Mar–Sep 2020). A total of 34,782 participants completed both waves (72.5% retention rate across years). Our study focuses on participants who completed either Time 10 or Time 11, meaning that our sample includes both participants that (a) completed both waves (34,782 participants) and (b) only completed one of the two waves (21,071 participants). Participants were sampled from the New Zealand electoral roll (see Sibley (2021)) and closely represent the New Zealand population in terms of socioeconomic status, age, and region of residence. However, women are overrepresented by approximately 12% (Stats NZ, 2018). For the current study, 11,289 women and 6,635 men in Time 10, and 26,683 women and 14,970 men in Time 11, completed the variables focused on in the current study (i.e., life events using the BISLE and gender) and are included in the analyses. As the current study draws on data embedded within the larger NZAVS survey, some participants may have chosen not to answer some questions or parts of the survey. Participants who did not complete the variables focused on in the current study were thus not included in the current analyses. Some participants may also return their surveys outside of the data collection period due to unforeseen circumstances. Therefore, participants who completed the survey outside the time period (Oct–Sep) covered by both waves were also excluded from the analyses. See section 1 in the Supplementary Material for further demographic characteristics. Supplementary Table 1 (see section 1 in Supplementary Material) shows that participants who completed all of the variables focused on in the current study and who completed Time 10 and/or Time 11 between October and September had comparable demographic characteristics, including similar proportions of ethnic groups and a similar average age.

### Measures

#### Life Events

The BISLE (Howard et al., 2022) asks participants to complete a checklist of 15 common life events (e.g., birth of a child, retired), followed by an open-ended question asking: “Have we missed anything important or would you like to provide more detail about your experiences?” Open-ended responses were then coded according to 590 specific life events (e.g., redundancy; Level 3) according to a detailed coding guide, and then collapsed into 141 broad life events categories (e.g., job loss; Level 2), which fall within 22 general life events domains (e.g., work; Level 1). The BISLE uses the life-change adaptation perspective and defines life events as significant changes that impact how people conduct their lives (Luhmann et al., 2012). The events included in the BISLE draw on previous inventories (e.g., SRRS; Holmes and Rahe, 1967) and include a range of positive and negative events, more specific events than prior inventories, and novel domains (e.g., discrimination, social issues; see Howard et al., 2022). More details on the BISLE, including the coding guide, is available in the Supplementary Material for the inventory paper by Howard et al. (2022) at: https://osf.io/75snb/. As the BISLE requires participants to self-generate events beyond the checklist of events provided, the inventory captures events that participants deem important in their own lives, rather than the objective occurrence of all events (for more information, see Howard et al., 2022). For the current study, coders were blind to the year responses were drawn.

Our analyses focus on the life event categories (Level 2) relevant to the economic, social, and well-being experiences of the pandemic. Table 1 shows these event categories (first column) along with specific events within each category (second column). Some relevant event categories were omitted from analyses due to their distributions being too low to be examined by gender and month: (1) relationship difficulties, (2) discrimination, (3) technology use, (4) positive lifestyle changes, (5) unemployment. After analyzing the proportion of reported life event categories during versus prior to the pandemic, we assess whether any differences are stable or variable across more specific life events within each category by examining up to three specific events within each category that meet a threshold of at least 100 overall frequency counts in both the 2018/2019 (Time 10) and 2019/2020 (Time 11) data collection waves.

**TABLE 1:** Event categories of interest and their specific events in the Broad Inventory of Specific Life Events (BISLE).

Life event category (*N*)	Specific life events (*N*)
Pandemic-related events (2,260)	*Pandemic/Epidemic* (1,744) Self contracted the virus (4) Family member or close other contracted the virus (7) *Experienced lockdown* (1,404) *Self directly impacted by pandemic/epidemic (financial, work)* (272) Family member or close other directly impacted by pandemic/epidemic (73) Pandemic/epidemic anxiety and/or uncertainty (71) Vaccine concerns (1) Got vaccinated (0) Lockdown loneliness (25) Started wearing a mask, observing social distancing, and/or use of PPE (18) Virus scare/test (7) Family member or close other had virus scare/test (0) Self-isolation (51) Family member or close other went into self-isolation (2) Change in alert levels (121) Issues with managed isolation facilities (1)
**Economic**	
Job loss (3,298)	Lost your job or had the principal earner in your household lose their job* (2,922) Fired at work (7) Redundancy (85) Partner made redundant (23) *Resigned from job* (358) Partner resigned from their job (28) Partner lost job (principal earner not defined) (36) Family member lost/quit job (not principal earner) (25)
Retirement (2,596)	Retired* (2,416) Semi-retired (87) Partner retired (89) Considering or planning retirement (72)
Employment changes (2,358)	Change in work hours or conditions (77) Change in responsibilities at work (62) Change to a different line of work (135) Change job (same line of work) (74) Increased workload/work hours (86) *Reduced/Lost work hours* (152) Working from home/working remotely (87) Business readjustment/restructuring (103) Promotion or pay rise (91) Began work (entered workforce) (85) Re-entered workforce (137) Stopped work (99) Partner began or re-entered work (25) Partner stopped work (20) Reduced pay or demoted (16) Started or bought own business (125) Became self-employed (54) Sold or lost business (68) Business struggling/struggling to keep business running (79) *New or change of job not further specified* (655) Partner changed job (91) Out of work (52) Partner out of work (9) *Looking for a job* (138) Essential worker (56) Started a second job (23) Work change not further specified (26) Other change in work (245)
Workplace issues (725)	Conflict at work (57) *Workplace harassment or bullying (directly affected)* (165) *Job insecurity* (122) *Stress (significant)* (188) Accident at work (19) Partner harassed or bullied at work (9) Trouble with boss (94) Felt unsafe at work (12) Work-life balance struggles (74) Workplace issue not further specified (10) Other workplace issue (115)
Financial concerns (485)	Decline in financial state (76) Bankruptcy (3) *Income decreased substantially (20% or more)* (118) Significant financial insecurity (52) *Financial difficulties* (139) Financial pressure (93) Went on a benefit (51) Wage subsidy and/or furloughed (13) Pay cut (permanent or temporary) (6) Issues with credits/refunds for canceled events and/or travel (1)
**Social**	
Relationship breakdown (2,327)	Breakdown of relationship (45) Got divorced* (346) Separated from your romantic partner/spouse* (1,982) Relationship break-up (76) Pending divorce (6) Cheated on partner (9) Partner cheated on you (22)
Family connection (481)	Family reunion (34) *Increase in time spent with family* (141) Improvement in relationship with family member (10) *Increased support from/to family and friends* (330) Family member entered relationship (9)
Family troubles (607)	Trouble with in-laws (7) *Trouble with family members* (166) Estrangement (43) Distancing family members (55) *Relationship breakdown, break-up or difficulties for a close family member* (133) *Isolation from family and friends* (168) Concerns about family member (102)
Traumatic interpersonal events (2,958)	Someone assaulted you, abused you, or attacked you* (2,448) Someone sexually harassed you* (664) Someone sexually assaulted you (14) Domestic violence (26) Family member attacked or assaulted (56) Family member experienced abuse (39) Bullied, stalked, or threatened (includes online) (62) Other traumatic interpersonal event (27)
**Well-being**	
Negative lifestyle changes (614)	Began negative personal habits (11) Partner or family member began negative personal habits (25) Stopped positive personal habits (41) *Less participation in social activities/recreation* (521) Worse sleeping habits (34)
Mental health (995)	Negative change in mental health (118) Positive change in mental health (60) Mental health problem or episode (113) *Mental health problem of family member* (225) Mental health problem of partner (55) Mental health problem of close friend (15) Negative change in partner’s mental health (8) Negative change in family member’s mental health (22) Positive change in partner’s mental health (3) Positive change in family member’s mental health (4) OCD (6) *Depression* (193) *Anxiety* (163) Suicidal thoughts (13) Fear of the unknown/uncertainty over future (86) Fear of contact with others/leaving the house (9) Attempted suicide (10) Family member attempted suicide (40) Partner attempted suicide (5) Close friend attempted suicide (6) Began counseling or therapy (93) Family member or partner began counseling or therapy (9)

Italicized specific events are those included in the analyses that meet the frequency count threshold of 100 overall counts in both annual waves of data collection (2018/2019 and 2019/2020). Events with an * next to them refer to the events provided in the checklist of 15 common events within the BISLE. Ns refer to the frequency count of participants who reported an event across both waves. As the event categories include a participant reporting at least one of the specific events, the sum of the frequency counts for the specific events may not equal the number stated at the event category level.

#### Gender

The NZAVS assesses gender with one open-ended question: “What is your gender?” Responses are coded into general identity categories (e.g., women, men, transgender, etc.). We focus on participants who identified as women or men.

### Analytic Overview

We first examined if the monthly proportions of women and men reporting economic, social, and well-being life events from the past year changed during the pandemic compared to the months and year before the pandemic. To do this, we conducted chi-squares to test if there were significant differences in proportions of each life event category shown in Table 1 reported across two blocks of time—pre-pandemic (Oct–Feb) and post-pandemic (Mar–Sep)—in the annual wave that the pandemic occurred (2019/2020) compared to the corresponding blocks of time in the annual wave prior to pandemic (2018/2019). If the pandemic impacted rates of life events, a significant chi-square should reveal an increase in number of events occurring from the onset of the pandemic (from March 2020, midway through the annual wave of data collection the pandemic occurred [2019/2020]).

Next, we conducted more specific examinations of differences in life events between each month in the annual wave when the pandemic occurred (2019/2020) compared to the same month in the prior annual wave (2018/2019). These comparisons do not reflect within-person changes but rather an examination of differences across pre-pandemic monthly averages compared to post-pandemic monthly averages to illustrate relative proportions in life events reported. If the pandemic was associated with an increase in relevant life events, these univariate chi-square tests within each month across years should reveal significant differences between proportions of events reported from March 2020 onward (compared to the same months in 2019). We theorized two potential patterns in the proportion of women and men reporting economic, social, and well-being events. First, given that participants report the life events experienced over the past 12 months, it is possible that an increase in the proportion of life events occurred as COVID-19 transmission emerged in New Zealand (March 2020) and this increase continued and potentially grew over the months of the pandemic, indicating a persistent change in experiencing (and thus reporting) of specific events. Alternatively, reports of relevant events may be greater at particular points in the pandemic when certain challenges were most salient for people (e.g., during lockdowns, returning to work, etc.).

Our results are provided in the context of the 4-tier Alert Level System that governed the restrictions placed on people in New Zealand during the pandemic (New Zealand Government, 2020, 2021). On 25 March 2020, New Zealand entered Level 4 national lockdown—the highest level of restriction, requiring people to stay at home except for essential movement. At Level 3, which occurred on 27 April 2020, people were still required to stay at home and most businesses remained closed, but restrictions eased including allowing up to 10 people gatherings for weddings and funerals. On 13 May 2020, Alert Level 2 allowed gatherings of up to 100 people and businesses were permitted to reopen with social distancing rules in place. On 08 June 2020, Alert Level 1 essentially meant life back to normal, with no social distancing rules or limits on social gatherings in place. However, a second community outbreak occurred in August 2020, which placed Auckland (the largest New Zealand city) back into Alert Level 3 (with some additional restrictions) on 12 August 2020. As above, challenges people faced due to the pandemic should be evident across the months of the pandemic irrespective of specific restrictions, but it is also possible that reports of relevant events may be greater at particular Alert Levels when certain challenges were most salient.

We conduct these tests for women and men separately because of systematic gender differences in reporting of events (see Davis et al. (1999), Dohrenwend (2006)). Men often under-report experienced events compared to women, with some scholars proposing that women are more able to detect and relay the occurrence of a significant life event (see Davis et al. (1999)). A prior analysis of NZAVS data from 2018/2019 also found that women, on average, reported more life events than men across several domains (Howard et al., 2022). These gender differences cannot be controlled for when using checklist and open-ended response to assess life events (see Dohrenwend (2006)) and so combining life events across women and men would provide an inaccurate picture of relative differences in life events across months. Moreover, although we cannot directly compare proportions of life events across women and men, prior reports examining the economic, social, and well-being impact of the pandemic indicate women have been disproportionately affected (UN Women, 2020).

## Results

### Pandemic Events

As pandemic events were not of relevance before 2020, open-ended responses from the annual wave before the pandemic (2018/2019) were blindly recoded alongside life events reports in the annual wave the pandemic began (2019/2020). Given that pandemic-related disruption occurred in New Zealand from March 2020, persistent reporting of pandemic events from this point onward would demonstrate that people were spontaneously reporting life events relevant to the challenges of the pandemic and thus provide evidence of the validity of our approach in comparing reported life events across years.

**FIGURE 2:**
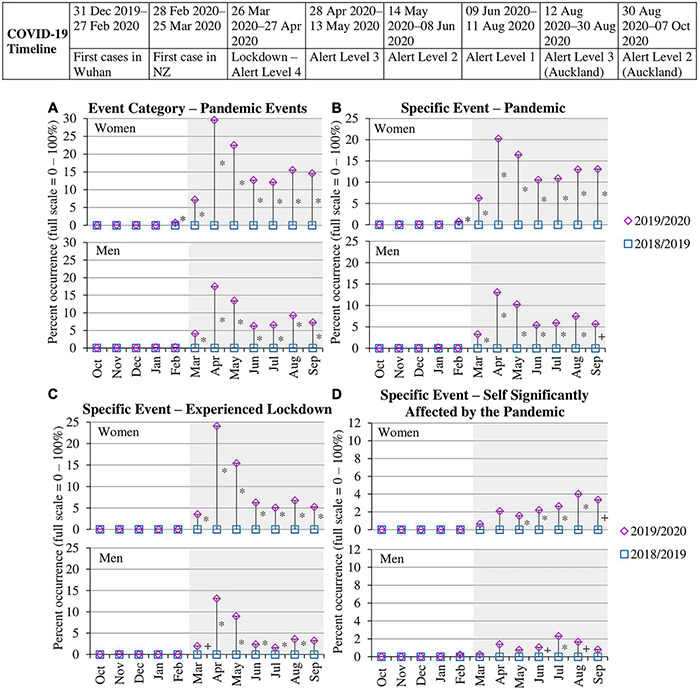
Percent occurrence of pandemic events category **(A)** and top specific events **(B–D)** for women and men in data collection wave prior to pandemic (Oct 2018–Sep 2019) vs. wave when pandemic occurred (Oct 2019–Sep 2020). * to the right of the line indicates a significant difference (*p* < 0.01) between the two time points within that month. + to the right of the line indicates a significant difference (*p* < 0.05) between the two time points within that month. Gray shading indicates the months of the pandemic occurring in New Zealand. Standard errors for each proportion across months for the two waves are provided inside each point.

Table 2 provides the average proportions for the pre- and post-pandemic time periods in 2018/2019 and 2019/2020 data collection waves. The proportion of women and men reporting pandemic events from March to September 2020 was substantially higher than the other time periods. Given the comparison year (2018/2019) had proportions of zero, inferential tests of this difference could not be conducted, but the obvious increase during the months of the pandemic clearly illustrate an uptick in these events during this time. To examine the specific pattern of life events that occurred in more detail, we conducted univariate chi-squares tests to examine differences in life events reported each month in the wave of data collection prior to the pandemic (2018/2019) versus the wave the pandemic occurred (2019/2020). Figure 2 shows the proportions of women and men reporting a pandemic event for each month (Oct–Sep) in 2018/2019 versus 2019/2020 collection waves. Figure 2 also graphs the same proportion for the top three specific events reported under this category: pandemic, experienced lockdown, self significantly affected by the pandemic. Significant chi-square tests indicating differences across years are indicated to the right of a month by * for differences significant at *p* < 0.01 or + for differences significant at *p* < 0.05.

**TABLE 2:** Proportions of women and men reporting pandemic events across Oct–Feb and Mar–Sep in 2018/2019 and 2019/2020.

	Women				Men			
	2018/2019		2019/2020		2018/2019		2019/2020	
Category (*N*) Specific events (*N*)	Pre-pandemic (Oct–Feb)	Pre-pandemic (Mar–Sep)	Pre-pandemic (Oct–Feb)	Post-pandemic (Mar–Sep)	Pre-pandemic (Oct–Feb)	Pre-pandemic (Mar–Sep)	Pre-pandemic (Oct–Feb)	Post-pandemic (Mar–Sep)
**Pandemic (2,260)** Pandemic (1,744) Lockdown (1,404) Self significantly affected (272)	0.00 0.00 0.00 0.00	0.00 0.00 0.00 0.00	0.08 0.07 0.02 0.00	**17.31** **13.25** **11.06** **2.08**	0.00 0.00 0.00 0.00	0.00 0.00 0.00 0.00	0.04 0.03 0.02 0.01	**9.58** **7.56** **5.49** **1.17**

*Bolded proportions indicate the time period that the COVID-19 pandemic occurred in New Zealand. Inferential tests could not be conducted as the comparison year (2018/2019) had proportions of zero. Ns refer to the frequency count of participants who reported an event across both waves. As the event categories include a participant reporting at least one of the specific events, the sum of the frequency counts for the specific events may not equal the number stated at the event category level.*

Demonstrating the validity of the life events inventory, there were no reported pandemic events reported by women and men in 2019. Moreover, both women and men began reporting pandemic events in March 2020 when the pandemic emerged in New Zealand and continued across the following months, ranging from 7.17% to 29.57% for women and 4.13% to 17.52% for men, all of which showed a significant increase from 2019. Reports of the specific events of ‘pandemic’ and ‘experienced lockdown’ also increased from March 2020 onward compared to the prior year. However, there was a slight delay in reports of ‘self significantly affected by the pandemic,’ which began in May for women and June 2020 for men and was then persistent through to August 2020. This pattern suggests that the personal effects of the pandemic were potentially not felt until the months following lockdown when New Zealanders were grappling with the aftermath as typical work and social activity resumed (for more details, see section 2 in Supplementary Material). These results show that women’s and men’s spontaneous reporting of pandemic life events changed in response to the conditions of the pandemic illustrating the validity of our approach in assessing differences in reported life events during versus prior to the pandemic. We applied the same analytic strategy to assess differences in economic, social, and well-being events.

### Economic Events

#### Job Loss

**FIGURE 3:**
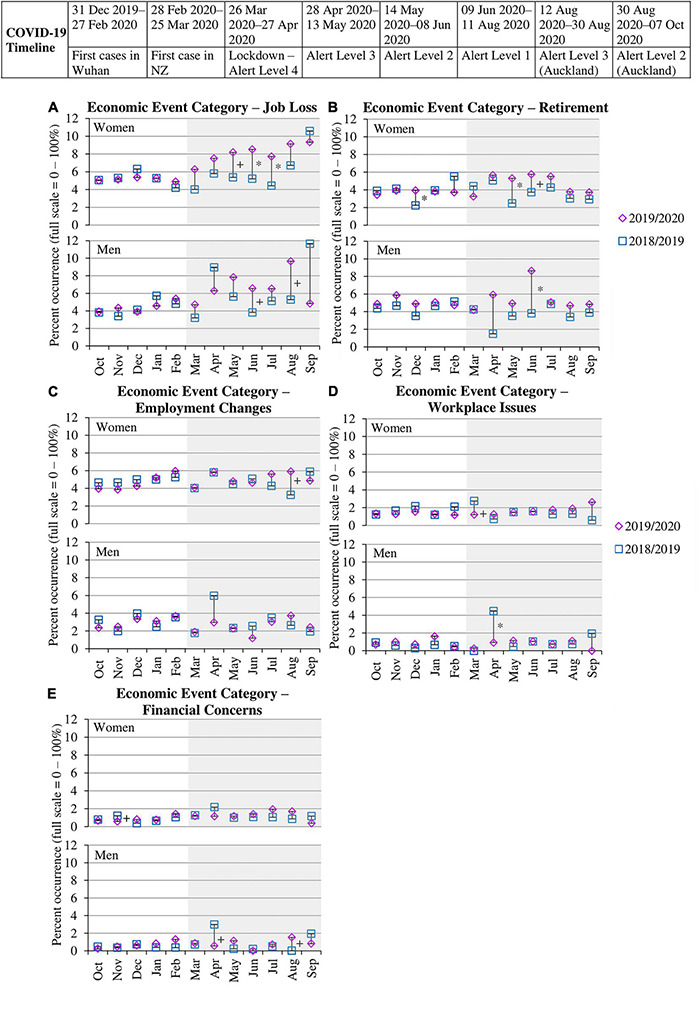
Percent occurrence of economic event categories **(A–E)** for women and men in data collection wave prior to pandemic (Oct 2018–Sep 2019) vs. wave when pandemic occurred (Oct 2019–Sep 2020). * to the right of the line indicates a significant difference (*p* < 0.01) between the two time points within that month. + to the right of the line indicates a significant difference (*p* < 0.05) between the two time points within that month. Gray shading indicates the months of the pandemic occurring in New Zealand. Standard errors for each proportion across months for the two waves are provided inside each point.

Table 3 shows a marked and significant increase in the proportion of women and men reporting job loss during the months of the pandemic (Mar–Sep 2020) compared to the other three time periods [women χ^2^(3) = 20.53, *p* < 0.001; men χ^2^(3) = 5.09, *p* = 0.024]. To examine the specific pattern of job loss from March 2020 onward, we conducted univariate chi-squares tests to examine differences in life events reported each month in the annual wave of data collection prior to the pandemic (2018/2019) versus the wave in which the pandemic occurred (2019/2020). As shown in Figure 3, women reported significantly higher rates of job loss from March to August 2020, but these rates were only significantly higher in May (8.19%), June (8.52%), and July (7.94%) of 2020 compared to the same months in 2019 (5.37%, 5.21%, 4.42%). These are the months following Level 4 and Level 3 national lockdown when restrictions eased, and employment activity could resume. A more variable pattern of differences across 2019 and 2020 emerged in men’s reporting of job loss, although the significant differences revealed that men reported significantly more job loss in June (6.58%) and August 2020 (9.66%) compared to the corresponding months of the previous year (3.82%, 5.28%). Analyses of the most commonly reported specific events under this category (“you or the principal earner lost your job” and “resignation”) revealed that the pattern in Figure 3 was specific to involuntary job loss, whereas voluntary job loss (i.e., resignation) revealed no significant differences across years (see Table 3 and sections 3.1, 3.6, and 3.7 in Supplementary Material).

**TABLE 3:** Proportions of women and men reporting economic events across Oct–Feb and Mar–Sep in 2018/2019 and 2019/2020.

	Women				Men			
	2018/2019		2019/2020		2018/2019		2019/2020	
Category (*N*) Specific events (*N*)	Pre-pandemic (Oct–Feb)	Pre-pandemic (Mar–Sep)	Pre-pandemic (Oct–Feb)	Post-pandemic (Mar–Sep)	Pre-pandemic (Oct–Feb)	Pre-pandemic (Mar–Sep)	Pre-pandemic (Oct–Feb)	Post-pandemic (Mar–Sep)
**Job loss (3,298)** Lost your job (2,922) Resigned from your job (358)	5.22 4.30 0.95	5.26 4.53 0.73	5.19 4.48 0.71	7.85** 7.15** **0.58**	4.38 3.96 0.43	5.14 4.52 0.46	4.08 3.65 0.44	6.64* 6.31** **0.24**
**Retirement (2,596)** Retired (2,416)	3.97 3.59	3.68 3.32	3.74 3.40	4.79** 4.53**	4.48 4.26	4.06 3.73	5.00 4.71	**5.40** **5.21**
**Employment changes (2,358)** Changed job (655) Reduced/Lost work hours (152) Looking for a job (138)	4.87 1.41 0.39 0.27	4.43 1.55 0.22 0.27	4.26 1.32 0.21 0.29	5.12** **1.00** 0.40** **0.28**	2.96 0.85 0.14 0.10	2.82 0.66 0.25 0.12	2.87 0.88 0.16 0.18	**2.51** **0.61** **0.24** **0.13**
**Workplace issues (725)** Workplace bullying (165) Stress (188) Job insecurity (122)	1.58 0.36 0.56 0.18	1.50 0.56 0.36 0.10	1.40 0.32 0.33 0.21	**1.50** **0.40** **0.36** 0.38*	0.64 0.10 0.24 0.05	0.83 0.00 0.29 0.08	0.80 0.10 0.17 0.15	**0.80** **0.15** **0.17** **0.26**
**Financial concerns (485)** Income decreased (118) Financial difficulties (139)	0.82 0.14 0.24	1.09 0.17 0.39	0.76 0.18 0.23	**1.33** **0.42** **0.35**	0.45 0.05 0.12	0.50 0.04 0.17	0.49 0.15 0.14	**0.80** **0.22** **0.19**

*Bolded proportions indicate the time period that the COVID-19 pandemic occurred in New Zealand. Ns refer to the frequency count of participants who reported an event across both waves. As the event categories include a participant reporting at least one of the specific events, the sum of the frequency counts for the specific events may not equal the number stated at the event category level.*

*Indicates a significant difference between the four time periods at p < 0.05.

**Indicates a significant difference between the four time periods at p < 0.01.

#### Retirement

As shown in Table 3, women showed a notable and significant increase in reported retirement during the months of the pandemic [Mar–Sep 2020; χ^2^(3) = 9.28, *p* = 0.002]. Conversely, men showed more variable proportions across time periods that were not significantly different from each other [χ^2^(3) = 1.11, *p* = 0.292]. As shown in Figure 3, comparing the specific pattern from March 2020 onward, women reported significantly higher rates of retirement in May (5.38%) and June (5.76%) 2020 compared to the same months of the previous year (2.50% and 3.74%). In contrast, reported retirement for men was more variable between years, with the only significant increase from 2019 occurring in June 2020 (8.67%). Analyses of the most frequently reported specific event within this category of “retired” revealed a similar pattern for women and men (see Table 3 and sections 3.2, 3.6, and 3.7 in Supplementary Material).

#### Employment Changes

As shown in Table 3, women showed a significant increase in reports of employment changes during the months of the pandemic [Mar–Sep 2020; χ^2^(3) = 8.46, *p* = 0.004], whereas men showed a more stable pattern of proportions across time periods that did not significantly differ [χ^2^(3) = 0.31, *p* = 0.576]. As shown in Figure 3, women and men showed a variable pattern of differences across 2018/2019 and 2019/2020 data collection waves from March 2020 onward, although women reported significantly more employment changes in August 2020 (5.92%) compared to August 2019 (3.25%) when Auckland went back into a regional lockdown, again halting normal employment activity. Analyses of the top three reported specific events under this category (“change of job,” “reduced/lost work hours,” and “looking for a job”) showed a similar variable pattern of differences (see Table 3 and sections 3.3, 3.6, and 3.7 in Supplementary Material).

#### Workplace Issues

Table 3 shows a stable pattern in the proportion of women and men reporting workplace issues across the four time periods, including during the months of the pandemic (Mar–Sep 2020), supported by non-significant chi-squares (*p*s < 0.05). Figure 3 shows little difference between years across the months of the pandemic for women and men, although women reported significantly less workplace issues in March 2020 (1.22%) compared to 2019 (2.74%) and men showed a significant decrease in April 2020 (0.93%) compared to the prior year (4.48%). Analyses of the most frequently reported specific events (“stress,” “job insecurity,” and “workplace harassment or bullying”) revealed no significant differences across years, except for a significant decrease in reports of stress among men in April 2020 compared to April 2019 and a significant increase in job insecurity among women in July 2020 compared to July 2019 (see Table 3 and sections 3.4, 3.6, and 3.7 in Supplementary Material).

#### Financial Concerns

Table 3 shows a stable pattern in the proportion of women and men reporting financial concerns across the four time periods, supported by non-significant chi-squares (*p*s < 0.05). Figure 3 further shows no significant differences across years for women and men, except than men showed a significant decrease in April 2020 (0.56%) compared to April 2019 (2.99%) and a significant increase in August 2020 (1.52%) compared to the previous year (0.00%). Analyses of the most commonly reported specific events (“decreased income” and “financial difficulties”) were reflective of this pattern of differences (see Table 3 and sections 3.5, 3.6, and 3.7 in Supplementary Material).

#### Summary of Economic Events

Involuntary job loss among women and men, as well as retirement among women, were events experienced or most salient during the months following lockdown when businesses attempted to continue employment activity with fewer resources. Employment changes among women were reported more during the second regional lockdown in Auckland, when normal employment activity was halted again. In contrast, workplace issues and financial concerns showed inconsistent differences, but hinted that (a) the lockdown temporarily removed some workplace stressors but women felt greater job insecurity following lockdown and (b) men were less concerned about finances during the initial nationwide lockdown, perhaps due to governmental wage assistance, but more concerned about finances later during the second regional lockdown.

### Social Events

#### Relationship Breakdown

**FIGURE 4:**
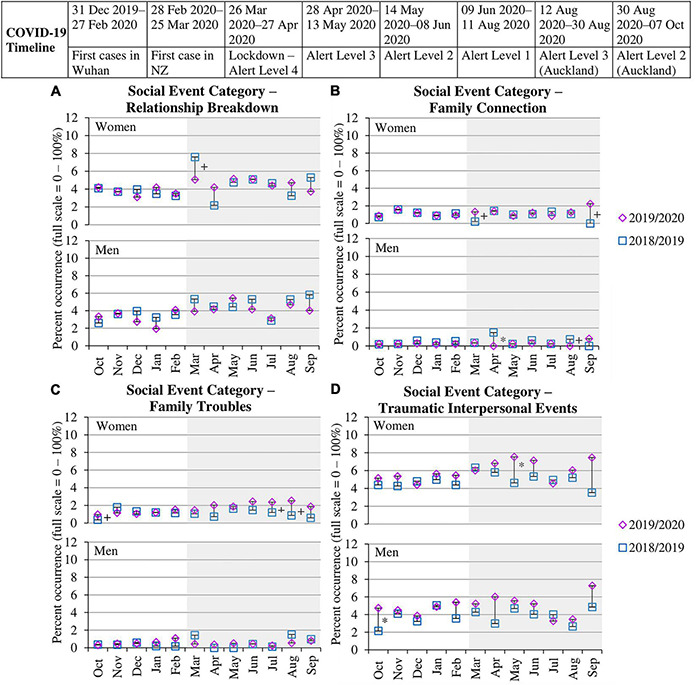
Percent occurrence of social event categories **(A–D)** for women and men in data collection wave prior to pandemic (Oct 2018–Sep 2019) vs. wave when pandemic occurred (Oct 2019–Sep 2020). * to the right of the line indicates a significant difference (*p* < 0.01) between the two time points within that month. + to the right of the line indicates a significant difference (*p* < 0.05) between the two time points within that month. Gray shading indicates the months of the pandemic occurring in New Zealand. Standard errors for each proportion across months for the two waves are provided inside each point.

Table 4 shows a variable pattern in the proportion of women and men reporting a relationship breakdown across the four time periods, supported by non-significant chi-squares (*p*s < 0.05). Figure 4 further shows no significant differences across years between March and September 2020 for women and men, except for a significant decrease among women in March 2020 (5.08%) compared to the year before (7.60%). Analyses of the most commonly reported specific events under this category (“separated from your spouse/romantic partner” and “got divorced”) showed that the pattern shown in Figure 4 was more relevant to a decrease in separation during the lockdown, whereas divorce revealed no significant differences across years (see Table 4 and sections 4.1, 4.5, and 4.6 in Supplementary Material).

**TABLE 4:** Proportions of women and men reporting social events across Oct–Feb and Mar–Sep in 2018/2019 and 2019/2020.

	Women				Men			
	
	2018/2019		2019/2020		2018/2019		2019/2020	
	
Category (*N*) Specific events (*N*)	Pre-pandemic (Oct–Feb)	Pre-pandemic (Mar–Sep)	Pre-pandemic (Oct–Feb)	Post-pandemic (Mar–Sep)	Pre-pandemic (Oct–Feb)	Pre-pandemic (Mar–Sep)	Pre-pandemic (Oct–Feb)	Post-pandemic (Mar–Sep)
**Relationship breakdown (2,327)** Separated from romantic partner/spouse (1,982) Got divorced (346)	3.70 3.10 0.56	4.87 4.12 0.78	3.71 3.11 0.53	**4.73** **4.02** **0.71**	3.32 2.84 0.55	4.35 3.69 0.66	3.13 2.72 0.48	**4.19** **3.70** **0.56**
**Family connection (481)** Increased support to/from family and friends (330) Increased time spent with family (141)	1.09 0.68 0.39	1.02 0.75 0.22	1.08 0.80 0.30	**1.21** **0.80** 0.39*	0.36 0.21 0.05	0.41 0.29 0.17	0.24 0.15 0.06	**0.21** **0.13** **0.06**
**Family troubles (607)** Trouble with family members (166) Isolation from family and friends (168) Relationship breakdown for family member (133)	1.15 0.32 0.17 0.42	1.24 0.49 0.15 0.32	1.06 0.37 0.14 0.28	2.04** **0.34** 1.03** **0.21**	0.33 0.10 0.05 0.10	0.50 0.12 0.04 0.21	0.46 0.17 0.10 0.08	**0.43** **0.07** **0.19** **0.09**
**Traumatic interpersonal events (2,958)** Someone assaulted, attacked, or abused you (2,448) Someone sexually harassed you (664)	4.58 3.56 1.26	5.11 4.27 1.24	4.96 3.89 1.27	**6.48** **5.05** 2.07**	3.63 3.32 0.43	4.02 3.60 0.54	4.43 4.09 0.44	**4.99** **4.56** **0.58**

*Bolded proportions indicate the time period that the COVID-19 pandemic occurred in New Zealand. Ns refer to the frequency count of participants who reported an event across both waves. As the event categories include a participant reporting at least one of the specific events, the sum of the frequency counts for the specific events may not equal the number stated at the event category level.*

*Indicates a significant difference between the four time periods at p < 0.05.

**Indicates a significant difference between the four time periods at p < 0.01.

#### Family Connection

Table 4 shows a variable pattern in the proportion of women and men reporting family connection events across the four time periods, supported by non-significant chi-squares (*p*s < 0.05). As shown in Figure 4, women showed no significant differences across years, apart from a significant increase in March (1.32%) and September (2.24%) 2020 compared to the previous year (0.21% and 0.00%). In contrast, men showed two significant decreases in April (0.00%) and August (0.00%) 2020 compared to the prior year (1.49% and 0.76%). Analyses of the most frequently reported specific events under this category (“increased support to and/or from family and friends” and “increased time spent with family”) revealed a variable pattern of proportions across years for women, but more stable proportions across years for men with one significant decrease in family support in August 2020 compared to August 2019 and one significant decrease in time spent with family in April 2020 compared to April 2019 (see Table 4 and sections 4.2, 4.5, and 4.6 in Supplementary Material).

#### Family Troubles

Table 4 shows a significant increase in the proportion of women reporting family troubles during the months of the pandemic [Mar–Sep 2020; χ^2^(3) = 9.11, *p* = 0.003], whereas men showed no significant differences [χ^2^(3) = 1.12, *p* = 0.291]. Figure 4 illustrates that women reported higher rates of family troubles between March and September 2020, but these were only significantly higher in July (2.38%) and August (2.54%) 2020 compared to the same months in 2019 (1.20% and 0.87%). These are the months when restrictions eased and people were able to reconnect with loved ones, before experiencing another lockdown in Auckland in late August 2020. Analyses of the most commonly reported specific events under this category (“trouble with family members,” “isolation from friends and family,” and “relationship breakdown for family member”) showed that the pattern in Figure 4 was specifically relevant to isolation from family and friends (see Table 4 and sections 4.3, 4.5, and 4.6 in Supplementary Material).

#### Traumatic Interpersonal Events

As shown in Table 4, the proportion of women reporting a traumatic interpersonal event increased during the months of the pandemic, but this increase was not significant [χ^2^(3) = 3.55, *p* = 0.060]. Men showed a stable pattern of traumatic events [χ^2^(3) = 0.00, *p* = 0.972]. Figure 4 shows that women only reported significantly higher rates of traumatic events in May 2020 (7.60%) compared to May 2019 (4.62%). Analyses of the most commonly reported specific events under this category (“someone assaulted you, abused you, or attacked you” and “someone sexually harassed you”) showed that the pattern in Figure 4 was specifically relevant to sexual harassment (see Table 4 and sections 4.4, 4.5, and 4.6 in Supplementary Material).

#### Summary of Social Events

Most social events overall showed inconsistent effects, with the pattern signaling that (a) separation decreased among women during lockdown, (b) connecting with family increased for women, but decreased for men, during the nationwide and regional lockdowns, and that (c) women reported more traumatic interpersonal events during lockdown. We also found that family troubles, particularly isolation from loved ones, was more salient or experienced for women during the months when restrictions eased, perhaps because reconnecting with loved ones was more difficult than expected or the sense of connection felt while being at home was lost when normal activities resumed.

### Well-Being Events

#### Negative Lifestyle Changes

**FIGURE 5:**
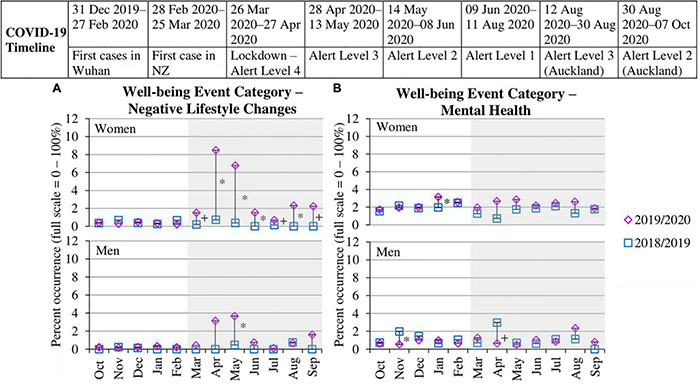
Percent occurrence of well-being event categories **(A,B)** for women and men in data collection wave prior to pandemic (Oct 2018–Sep 2019) vs. wave when pandemic occurred (Oct 2019–Sep 2020). * to the right of the line indicates a significant difference (*p* < 0.01) between the two time points within that month. + to the right of the line indicates a significant difference (*p* < 0.05) between the two time points within that month. Gray shading indicates the months of the pandemic occurring in New Zealand. Standard errors for each proportion across months for the two waves are provided inside each point.

As shown in Table 5, women reported greater negative lifestyle changes during the months of the pandemic [Mar–Sep 2020; χ^2^(3) = 269.67, *p* < 0.001], whereas men did not [χ^2^(3) = 43.50, *p* = 0.169]. Figure 5 shows that women began reporting negative lifestyle changes in March 2020 when the pandemic emerged in New Zealand and continued across the following months, ranging from 0.71% to 8.48%, all of which showed a significant increase from 2019. In contrast, men showed a more variable pattern, with only one significant increase May 2020 (3.67%) compared to the previous year (0.47%). The top specific event of ‘less participation in social activities or recreation’ showed the same pattern as Figure 5 (see Table 5 and sections 5.1, 5.3, and 5.4 in Supplementary Material). Overall, the pattern indicates that negative lifestyle changes, particularly less social activities, was persistently experienced or salient for women during the pandemic.

**TABLE 5:** Proportions of women and men reporting well-being events across Oct–Feb and Mar–Sep in 2018/2019 and 2019/2020.

	Women				Men			
	
	2018/2019		2019/2020		2018/2019		2019/2020	
Category (*N*) Specific events (*N*)	Pre-pandemic (Oct–Feb)	Pre-pandemic (Mar–Sep)	Pre-pandemic (Oct–Feb)	Post-pandemic (Mar–Sep)	Pre-pandemic (Oct–Feb)	Pre-pandemic (Mar–Sep)	Pre-pandemic (Oct–Feb)	Post-pandemic (Mar–Sep)
**Negative lifestyle changes (614)** Less participation in social activities or recreation (521)	0.46 0.18	0.17 0.12	0.41 0.19	3.98** 3.81**	0.07 0.07	0.17 0.12	0.22 0.10	**1.51** 1.41**
**Mental health (995)** Mental health problem of family member (225) Depression (193) Anxiety (163)	1.97 0.52 0.41 0.29	1.75 0.46 0.41 0.41	2.00 0.61 0.34 0.25	2.44* **0.38** **0.28** **0.51**	1.14 0.14 0.43 0.17	0.91 0.17 0.25 0.25	0.79 0.12 0.25 0.10	**1.08** **0.15** **0.28** **0.17**

*Bolded proportions indicate the time period that the COVID-19 pandemic occurred in New Zealand. Ns refer to the frequency count of participants who reported an event across both waves. As the event categories include a participant reporting at least one of the specific events, the sum of the frequency counts for the specific events may not equal the number stated at the event category level.*

*Indicates a significant difference between the four time periods at p < 0.05.

**Indicates a significant difference between the four time periods at p < 0.01.

#### Mental Health

As shown in Table 5, women reported greater mental health events during the months of the pandemic [Mar–Sep 2020; χ^2^(3) = 4.59, *p* = 0.032], whereas men did not [χ^2^(3) = 2.71, *p* = 0.100]. As shown in Figure 5, although overall proportions of women reporting mental health events increased during the months of the pandemic, the specific differences across years for each month revealed no significant differences from March to September 2020. Men also revealed no significant differences across years, except that April 2020 (0.65%) significantly decreased compared to April 2019 (2.99%). Analyses of the top three reported specific events under this category (“mental health problem of family member,” “depression,” and “anxiety”) showed a pattern of differences reflective of those in Figure 5 (see Table 5 and sections 5.2, 5.3, and 5.4 in Supplementary Material).

#### Summary of Well-Being Events

Women reported more negative lifestyle changes, particularly a reduction in social activities or recreation, during the pandemic. Although consistently higher from March 2020 onward compared to the prior year, proportions were substantially greater during the nationwide lockdown when women were confined to their home and unable to meet with non-resident loved ones or do usual recreational activities. In contrast, mental health remained relatively unchanged pre- versus post-pandemic, with the overall pattern perhaps weakly signaling that men may experience fewer mental health events during lockdown.

## Discussion

The current study examined changes in reports of economic, social, and well-being life events among women and men during the first 7 months of the COVID-19 pandemic compared to the year prior to the pandemic. We leveraged large-scale data from two annual waves of the NZAVS collected prior to (Oct 2018–Sep 2019; average *N* per month = 1,520) and during (Oct 2019–Sep 2020; average *N* per month = 3,551), the pandemic. Providing evidence of the validity of our examination of differences in life events pre- and post-pandemic, reports of pandemic events were consistently higher in the months of the pandemic (Mar–Sep 2020) compared to zero proportions in 2019.

Economic, social, and well-being events pre- and post-pandemic revealed larger and more consistent changes for economic events. Aligning with and extending prior research (e.g., Park et al., 2020), women and men reported increased job loss in the months following the nationwide lockdown relative to the same months in the prior year. Women also reported increased retirement in the months following national lockdown, as well as increased employment changes when stricter restrictions were reintroduced during a regional outbreak in Auckland (New Zealand’s biggest city). These findings also align with national New Zealand data that shows an increase in unemployment in the September 2020 quarter following New Zealand’s national lockdown (Stats NZ, 2020b). However, monthly averages of workplace issues and financial concerns did not reliably differ from the pre-pandemic baseline, contrasting findings from cross-sectional studies (e.g., Jean-Baptiste et al., 2020). Yet, our finding that women showed greater job insecurity following lockdown aligns with prior research (e.g., Park et al., 2020). In sum, increased reports of some economic events—particularly job loss and retirement—occurred following lockdown and women were disproportionately represented in these changes (see also Azcona et al. (2020) and UN Women (2020)).

Most social events, including relationship breakdown and family connection events, did not differ reliably from pre-pandemic levels, contrasting prior research (e.g., Whitehead and Torossian, 2021). However, supporting and extending existing cross-sectional data (e.g., Jean-Baptiste et al., 2020; Kujawa et al., 2020), women reported an increase in family troubles—particularly isolation from loved ones—during the pandemic. However, counter-intuitively, this increase occurred when restrictions eased, perhaps because reconnecting with loved ones was more difficult than expected or the sense of connection felt while being at home was lost when normal activities resumed. Overall, our findings suggest that social events were less affected by the pandemic than economic events, but women experienced the burden of feeling isolated from loved ones.

Women also showed the most changes in monthly proportions of reported negative lifestyle changes pre- versus post-pandemic. Specifically, women persistently reported increased negative lifestyle changes—particularly lower social activities and recreation—relative to pre-pandemic, with proportions particularly high during nationwide lockdown when people were confined to their home. This corroborates Entringer and Gosling’s (2021) findings on loneliness during the pandemic, including the higher risk of loneliness among women (see also Bu et al. (2020)). In contrast, monthly trends of reported mental health events were relatively unchanged pre- versus post-pandemic, corroborating findings supportive of average resilience across the pandemic (Aknin et al., 2021). These findings reflect the diverging patterns of risk and resilience seen across all our examined events, in which women bore the burden of increased negative life events (see Azcona et al. (2020)), while many events remained relatively unchanged (see Davis et al. (2021)).

### Theoretical Implications

Our research makes important advances in understanding the economic, social, and well-being repercussions of the pandemic. Extending prior cross-sectional research conducted early in the pandemic (e.g., Jean-Baptiste et al., 2020; Kujawa et al., 2020), our findings indicate that only a select few events—particularly job loss and negative lifestyle changes—reliably increased during the pandemic compared to pre-pandemic levels from the prior year. Furthermore, the burden of some increased negative events endured over time (negative lifestyle changes among women) whereas others occurred at specific points in the pandemic (e.g., job loss and retirement increased after lockdown). Lastly, our findings signal that women were disproportionately affected by increased negative events (see also Azcona et al. (2020) and UN Women (2020)).

Our study also advances existing knowledge on the impact of the COVID-19 pandemic by highlighting resilience to many potential negative life events. Many prior studies focus on identifying the stressors people have experienced due to the pandemic (e.g., Park et al., 2020). However, this narrow focus means that limited studies explore possible positive or resilient outcomes (e.g., Davis et al., 2021; Jenkins et al., 2021). Our study indicates that the challenges of the pandemic have not resulted in marked pre- and post- pandemic differences in monthly trends of reported life events, including financial concerns, workplace stressors, and mental health events. Thus, our findings provide further evidence for resilience during the pandemic across a range of different life domains that vary in severity and valence (e.g., traumatic interpersonal events to connecting with family).

Our findings also have important implications for assessing reported life events. The findings provide evidence for the validity of the BISLE as a useful measurement tool by indicating that people spontaneously report relevant events in their lives using the BISLE. Our findings also suggest that participants are likely to report events that are salient when they complete the survey, but those events may not reach a threshold of reporting as a life event once their relevance diminishes (Funch and Marshall, 1984; Pachana et al., 2011). For example, women reported high increases in negative lifestyle changes across the prior year when they were completing the survey during lockdown and thus daily activities and routines were halted, but reports of lifestyle changes across the year soon reduced when restrictions eased.

Given that participants likely only reported events salient and deemed important in their own lives at the time of completing the survey, the implications of the results may need to be considered with caution. For example, our results suggest that reports of mental health events for women and men did not reliably differ pre- and post-pandemic. Although most participants who received a mental health diagnosis (e.g., depression or anxiety disorders) will most likely report this as a significant event, some participants may decide that general mood changes are not a significant life event, and thus not spontaneously report a change in mood as a life event using the BISLE. Yet, our findings regarding mental health corroborate with other research conducted by Sibley et al. (2020), who found little change in distress pre- and post-lockdown in New Zealand using a more general measure of psychological distress. Future research using and comparing different methods, such as including both life events and more general psychological measures, will elucidate whether different types of measurement lead to different conclusions regarding changes in reported or perceived psychological functioning and well-being before and during the pandemic.

### Implications for Policy

Although we identified important changes pre- and post-pandemic for certain events, including job loss and negative lifestyle changes, the monthly proportions of these events were overall quite small. These small proportions may be due, in part, to the use of open-ended responses by the BISLE which require participants to self-generate and report events that they deem important in their own lives. Therefore, many of the estimates produced using the BISLE are conservative (see Howard et al. (2022)). The small monthly proportions as well as the small number of changes in life events may also be due to the current study examining the first 7 months of the pandemic. It may be that the repercussions of the pandemic were not fully realized during these first 7 months of the pandemic, but the salience and impact of changes in life events became more pronounced and/or accumulated across time as the pandemic has continued for 2 years at the time of writing. That said, our study examining the first 7 months of the pandemic covered unprecedented societal shifts that altered people’s daily routines, such as national lockdowns, providing important insight into how the pandemic was associated with changes in reported life events that can inform future policy and research as the pandemic continues over time.

It is particularly notable that we found significant increases in certain negative life events pre- and post-pandemic which, although small, might provide early indicators of the impact of the pandemic that can have important long-term consequences for overall public mental health and resources. For example, Chandola et al. (2020) longitudinal analysis of the effect of lockdown-related stressors on psychological distress revealed that social isolation and loneliness—the most prevalent negative lifestyle change in the current study—was the strongest predictor of increased psychological distress. Furthermore, people who reported experiencing job loss and unemployment were two times more likely to report higher psychological distress. Therefore, the small but significant number of people (particularly women) reporting increased negative life events may be especially susceptible to the ensuing detrimental consequences and lead to greater pressure on mental health services over time (see Gruber et al. (2020)).

In highlighting the life events that were reported more during the pandemic compared to pre-pandemic baseline rates, our study can inform the development of targeted policy and resource distribution to ensure communities are adequately supported during the pandemic (Gruber et al., 2020). For example, our findings that women and men were most vulnerable to job loss in the period after lockdown when government-assisted wage subsidies cease, and businesses attempt to re-start employment activity with fewer resources, suggests that providing more support to businesses to sustain employment during the months following lockdown is needed. Our findings also signal that the development of gender-responsive policy is required, including (a) ensuring equal access to job opportunities for women, (b) equal resources across employment types and sectors that include more women (e.g., hospitality, part-time workers), and (c) support for women to help balance work and greater family duties (Azcona et al., 2020). For example, governments could provide grants specifically targeted for women-owned businesses to reduce the likelihood of business closures, as well as provide income support for women working in sectors severely impacted by COVID-19 (e.g., hospitality) along with paid leave for women acting as primary caregivers (Azcona et al., 2020). Governments could also ensure access to important services for women, including family planning, equal wages, and education during the acute stages of the pandemic as well as during pandemic recovery (Azcona et al., 2020).

### Caveats and Future Directions

People in New Zealand, like other countries, have experienced numerous challenges throughout the pandemic, including mandatory lockdowns that created widespread economic challenges (employment/financial loss), social loss (confinement/isolation), and potential threats to well-being. Yet, the New Zealand government’s rapid response meant COVID-19 was successfully contained for much of the first year of the pandemic (Baker et al., 2020), which may partly explain why many examined events did not differ reliably from pre-pandemic baseline trends. New Zealand data also may not generalize to other countries more severely impacted by the pandemic (e.g., North America). Research conducted early in the pandemic hints at cross-country differences, with perceived stress greater in Western European countries, such as the United Kingdom (see Lieberoth et al. (2021)), as well as in countries more severely impacted by the pandemic (Kowal et al., 2020). Future research should examine reported life events across countries that are differentially affected by the pandemic to examine whether these effects are context-specific or whether universal patterns emerge.

Our analytic approach relied on making multiple comparisons across the first 7 months of the pandemic for each event, increasing the possibility of incorrectly rejecting the null hypothesis (i.e., greater likelihood of false positive effects). Such large comparisons were required for the exploratory nature of the current study, but future research would benefit from more targeted analyses that reduce multiple testing bias. Second, our comparisons do not reflect within-person changes but rather an examination of differences in monthly averages pre- versus post-pandemic in large groups from a nationally representative panel study. As we found important patterns of differences pre- and post-pandemic at the between-group level, future research should replicate our findings at the within-person level to see if similar patterns of changes are found when following the same people before and during the pandemic. Third, we interpreted our findings as indicating that women experienced more negative life events during the pandemic, but it is possible that this reflects women being more likely to report events than men (see Davis et al. (1999)). We tackled this potential caveat by only making pre- versus post-pandemic comparisons within each gender (i.e., 2019 versus 2020 for women) and so any differences in reported events are relevant to both the pre-pandemic benchmarks as well as during the pandemic. Using this approach, our results indicate that women showed differences in proportions between pre- and post-pandemic time periods, whereas there were little differences in pre- and post-pandemic trends observed for men.

Our findings indicate that women are disproportionately affected by increased negative life events during the pandemic. However, we did not examine differences across other groups that may be more likely to experience changes in negative life events (e.g., age cohorts, ethnic groups). For example, research highlights that, compared to older adults, younger people are at greater risk of negative mental health outcomes, including higher depressive symptoms, during the pandemic (see Palgi et al. (2020), Gozansky et al. (2021), and Valiente et al. (2021)). During the COVID-19 pandemic in particular, Rossi et al. (2021a) found that older adults had higher resilience, reducing their vulnerability to the negative mental health outcomes compared to younger adults. The negative outcomes experienced by younger people may be due to greater economic concerns regarding the pandemic (Gozansky et al., 2021). The current study provides the foundation to examine potential differential patterns of risk and resilience in reported economic, social, and well-being life events in other groups beyond gender. Future investigations might find differential patterns of changes in life events across different social groups based on the specific challenges faced by those groups.

The primary focus of our study was to provide an exploratory analysis of changes in reports of economic, social, and well-being life events during the pandemic compared to the same time period before the pandemic began. Given the established link between life events and psychological maladjustment (e.g., greater depressive symptoms; see Hammen (2005); Gruber et al. (2020)) and research evidence that pandemic stressors increase the risk of psychological distress (see Chandola et al. (2020)), future research should further examine the impact of increasing reports of negative life events on mental health outcomes. Building on the key findings of the current study, a valuable direction for future research involves whether the changes in job loss and social isolation during the pandemic have differential impacts on psychological distress. Research by Chandola et al. (2020) suggest that social isolation is one of the strongest predictors of psychological distress during the pandemic. Comparing changes in isolation compared to job activity may thus provide important insights into potential domain-specific effects of life events during the pandemic. In addition, as our study highlights that the burden of increased negative life events appears more heavily borne by women, future research should incorporate protective or risk factors (e.g., personal resilience, previous adversity, age) that may mitigate or exacerbate the association between life events and mental health during the ongoing COVID-19 pandemic. Overall, the current study provides important and informative data on changes in life events pre- versus post-pandemic that provide a valuable foundation for future research to understand the repercussions of the pandemic on mental health and other key outcomes (job security, social well-being, gender equality).

The COVID-19 pandemic continues to cause global disruption more than 18 months on from its emergence. Therefore, although our study advances extant research by examining changes in monthly averages of reported life events for the first 7 months of the pandemic in New Zealand, further research that documents the long-term impacts and after-effects of COVID-19 over coming years is needed (see also Lieberoth et al. (2021)). It may be that the increase in potential negative events plateaus as people get used to the ‘new normal’ of the pandemic or that negative events continue to increase as resources (and resilience) diminish over time from ongoing disruptions. Long-term data that follows the pandemic over upcoming years may also allow researchers to examine the after-effects of the pandemic, including how countries re-open and recover (see Lieberoth et al. (2021)).

## Conclusion

The current study examined changes in reported economic, social, and well-being life events among women and men during the first 7 months of the COVID-19 pandemic (Mar–Sep 2020) compared to the same months in the year preceding the pandemic. Our analyses utilized monthly averages in cross-sectional national probability data from two annual waves of the NZAVS, including the 2018/2019 wave prior to the pandemic (*N* = 17,924; average *N* per month = 1,520) and the 2019/2020 wave when the pandemic occurred in New Zealand (*N* = 41,653; average *N* per month = 3,551). In doing so, our study advanced the extant cross-sectional research conducted at the start of the pandemic by examining pre- versus post-pandemic changes in the monthly trend of reported life events. Validating our approach to compare life events pre- and post-pandemic and indicating that people spontaneously report relevant events in their lives, analysis of pandemic-related events confirmed that people persistently reported more pandemic events during the pandemic compared to zero proportions in the prior year.

Applying this approach to examine changes in economic, social, and well-being events revealed that monthly reports during the pandemic centered on a few events, particularly job loss and negative lifestyle changes. People (particularly women) reported increased economic events—including job loss and retirement—in the months following national lockdown relative to the same months the year earlier. Women also experienced an increase in family troubles—particularly isolation from loved ones—when restrictions eased, as well as an increase in negative lifestyle changes—in particular fewer social activities and recreation—that persisted throughout the first 7 months of the pandemic, compared to the prior year. However, many other life events, such as mental health, financial concerns, and relationship breakdowns, appeared largely unaffected by the pandemic in New Zealand and did not differ reliably from the pre-pandemic monthly baseline.

The overall pattern of results highlights resilience to many potential negative life events in New Zealand. This pattern may indicate a general resilience in the face of the pandemic as shown in other countries, or may be specific to the context of New Zealand’s rapid initial response to COVID-19 that successfully contained the first waves of the virus. Nonetheless, the pandemic did not affect everyone equally, with women disproportionately represented in increased life events during the pandemic. As significant life disruptions continue more than 18 months since the pandemic emerged, our findings provide valuable insight into the changes in life events that accompanied the first 7 months of the pandemic. These early changes have important implications for understanding the impact of the pandemic on people’s lives, such as the need for targeted gender-responsive policy and resource distribution to mitigate any potential negative impacts of life events on mental health, gender equality, and job security, occurring due to the pandemic.

## Data Availability Statement

The datasets presented in this article are not readily available because ethical restrictions and the need to protect the confidentiality of study participants prevent public deposition of raw data. The data described in this manuscript are part of the New Zealand Attitudes and Values Study (NZAVS). Full copies of the NZAVS data files are held by all members of the NZAVS management team and advisory board. A de-identified dataset containing the variables analyzed in this manuscript is available upon request from the corresponding author, or any member of the NZAVS advisory board for the purposes of replication or checking of any published study using NZAVS data. The full statistical standard for life events in the NZAVS (with coding details and generalized examples of responses) is provided under the supplementary information for the inventory article (Howard et al., 2022) available at https://osf.io/75snb/. The Mplus syntax used to test all models reported in this manuscript are available on the NZAVS website: www.nzavs.auckland.ac.nz (see also: https://osf.io/75snb/). Requests to access the datasets should be directed to CS, c.sibley@auckland.ac.nz.

## Ethics Statement

The studies involving human participants were reviewed and approved by The University of Auckland Human Participants Ethics Committee (Reference Number: 014889). The patients/participants provided their written informed consent to participate in this study.

## Author Contributions

CH conceptualized the study, performed the statistical analysis, wrote the manuscript, and performed any manuscript revision. All authors contributed to the design of the study, including the analytic strategy. CS organized the database and funding for the study. CS and NO provided supervision and extensive feedback on the manuscript.

## Conflict of Interest

The authors declare that the research was conducted in the absence of any commercial or financial relationships that could be construed as a potential conflict of interest.

## Publisher’s Note

All claims expressed in this article are solely those of the authors and do not necessarily represent those of their affiliated organizations, or those of the publisher, the editors and the reviewers. Any product that may be evaluated in this article, or claim that may be made by its manufacturer, is not guaranteed or endorsed by the publisher.

## Footnotes

1
www.nzavs.auckland.ac.nz

2
https://osf.io/75snb/

## Supplementary Material

The Supplementary Material for this article can be found online at: https://www.frontiersin.org/articles/10.3389/fpsyg.2022.829643/full#supplementary-material. Online Supplementary Material can also be found in the supporting information OSF for the NZAVS at https://osf.io/75snb/.
