# Supplementary material for: Monthly Trends in the Life Events Reported in the Prior Year and First Year of the COVID-19 Pandemic in New Zealand
Source: Front Psychol. 2022 Mar 11;13:829643. doi: 10.3389/fpsyg.2022.829643 (PMC8963340; doi:10.3389/fpsyg.2022.829643)

Online Supplemental Material

Table of Contents

1 Demographic Characteristics 3
2 Analyses of Top Specific Events under the Pandemic Event Category 3
2.1 Pandemic 3
2.2 Experienced Lockdown 4
2.3 Self Significantly Affected by the Pandemic 4
2.4 Summary 4
3 Analyses of Top Specific Events under the Economic Events Categories 4
3.1 Job Loss 4
3.1.1 You or the Principal Earner Lost your Job 4
3.1.2 Resigned from your Job 5
3.2 Retirement 5
3.2.1 Retired 5
3.3 Employment Changes 5
3.3.1 Change of Job 5
3.3.2 Reduced/Lost Work Hours 5
3.3.3 Looking for a Job 5
3.4 Workplace Issues 6
3.4.1 Stress (Significant) 6
3.4.2 Workplace Harassment or Bullying 6
3.4.3 Job Insecurity 6
3.5 Financial Concerns 6
3.5.1 Decreased Income 6
3.5.2 Financial Difficulties 6
3.6 Summary 6
3.7 Figure S1. 7
4 Analyses of Top Specific Events under the Social Events Categories 9
4.1 Relationship Breakdown 9
4.1.1 Separated from your Spouse/Romantic Partner 9
4.1.2 Got Divorced 10
4.2 Family Connection 10

4.2.1 Increased Support to and/or from Family and Friends 10
4.2.2 Increased Time Spent with Family 10
4.3 Family Troubles 10
4.3.1 Trouble with Family Members 10
4.3.2 Isolation from Friends and Family 10
4.3.3 Relationship Breakdown for Family Member 11
4.4 Traumatic Interpersonal Events 11
4.4.1 Someone Assaulted You, Abused You, or Attacked You 11
4.4.2 Someone Sexually Harassed You 11
4.5 Summary 11
4.6 Figure S2. 12
5 Analyses of Top Specific Events under the Well-Being Events Categories 14
5.1 Negative Lifestyle Changes 14
5.1.1 Less Social Activities or Recreation 14
5.2 Mental Health 14
5.2.1 Mental Health Problem of a Family Member 14
5.2.2 Depression 14
5.2.3 Anxiety 14
5.3 Summary 14
5.4 Figure S3. 15

1 Demographic Characteristics

Table S1. Demographic characteristics for participants who completed all of the variables focused on in the current study and who completed Time 10 and/or Time 11 of the New Zealand Attitudes and Values Study during the time period covered by both waves (Oct–Sep)

	**Time 10**		**Time 11**	
**Demographics**	**Mean (SD)**	**% (*n*)**	**Mean (SD)**	**% (*n*)**
Age	52.50 (13.57)		52.19 (13.81)	
Gender				
Women		62.98 (11,289)		64.06 (26,683)
Men		37.02 (6,635)		35.94 (14,970)
Ethnicity				
NZ European		90.84 (16,282)		92.73 (38,623)
Māori		11.45 (2,052)		10.03 (4,178)
Pacific Island		2.25 (404)		2.65 (1,104)
Asian		4.17 (747)		4.40 (1,833)
Other/did not report		2.40 (430)		2.55 (1,061)
Employed		76.64 (13,730)		75.98 (31,539)
Born in NZ		79.54 (14,243)		78.16 (32,488)
In a serious romantic relationship		75.34 (13,277)		74.79 (30,827)
Parent		75.31 (13,486)		73.47 (30,587)
Identified with a religious or spiritual group		36.50 (6,521)		33.75 (13,969)

2 Analyses of Top Specific Events under the Pandemic Event Category

2.1 Pandemic

As shown in Table 2 (see main text), the proportion of women and men reporting the specific event of ‘pandemic’ from Mar–Sep 2020 was substantially higher than the other time periods. Given the comparison year (2018/2019) had proportions of zero, inferential tests of this difference could not be conducted, but the obvious increase during the months of the pandemic clearly illustrate an uptick in this event. As shown in Figure 2 (see main text), both women and men began reporting the specific event of ‘pandemic’ in March 2020 when the pandemic emerged in New Zealand and continued across the following months, ranging from 6.25% to 20.22% for women and 3.26% to 13.07% for men, all of which showed a significant increase from 2019.

2.2 Experienced Lockdown

As shown in Table 2 (see main text), the proportion of women and men reporting the specific event of ‘experienced lockdown’ from Mar–Sep 2020 was substantially higher than the other time periods. Given the comparison year (2018/2019) had proportions of zero, inferential tests of this difference could not be conducted, but the obvious increase during the months of the pandemic clearly illustrate an uptick in this event. As shown in Figure 2 (see main text), both women and men began reporting experiencing lockdown in March 2020, when New Zealand went into a mandatory national lockdown that halted all activity except for essential movement. This continued across the following months, with proportions ranging from 3.51% to 24.09% for women and 1.59% to 13.16% for men, all of which showed a significant increase from 2019 (except for September among men).

2.3 Self Significantly Affected by the Pandemic

As shown in Table 2 (see main text), the proportion of women and men reporting the specific event of ‘self significantly affected by the pandemic’ from Mar–Sep 2020 was higher than the other time periods. Given the comparison year (2018/2019) had proportions of zero, inferential tests of this difference could not be conducted, but the obvious increase during the months of the pandemic clearly illustrate an uptick in this event. As shown in Figure 2 (see main text), there was a slight delay in both women and men women and men reporting being significantly affected by the pandemic, which began in May for women and June 2020 for men and was then persistent through to August 2020, with proportions ranging from 1.58% to 4.02% for women and 1.05% to 2.32% for men, all of which showed a significant increase from 2019 (except for September among men).

2.4 Summary

The pattern of results suggest that the most frequently reported pandemic-related events were consistently either more salient or more experienced throughout the pandemic. In particular, we found that women and men persistently reported higher rates of the specific events ‘pandemic’ and ‘experienced lockdown’ from March 2020 onwards, suggesting that these events were consistently salient for people during the pandemic. On the other hand, we found that women and men reported being significantly affected by the pandemic in the months following lockdown onwards. This suggests the personal effects of the pandemic were potentially not felt until the months following lockdown when New Zealanders were grappling with the aftermath of lockdowns as typical work and social activity resumed. Interestingly, rates of the specific event ‘self significantly affected by the pandemic’ were much lower overall than those reporting the events of ‘pandemic’ and ‘experienced lockdown’. Overall, this suggests that the pandemic and lockdowns were of ongoing relevance to women and men throughout the pandemic.

3 Analyses of Top Specific Events under the Economic Events Categories

3.1 Job Loss

3.1.1 You or the Principal Earner Lost your Job

Table 3 (see main text) shows a marked increase in the proportion of women and men reporting involuntary job loss during the months of the pandemic (Mar–Sep 2020) compared to the other three time periods (women ($\chi^2(3) = 18.21$, $p < .001$; men ($\chi^2(3) = 7.47$, $p = .006$). As shown in Figure S1, women reported significantly higher rates of job loss from March to August 2020, but these rates

were only significantly higher in March (5.37%), May (7.60%), June (7.71%), and July (7.32%) of 2020 compared to the same months in 2019 (3.17%, 4.74%, 4.67%, 3.52%). These are primarily the months following Level 4 and Level 3 national lockdown when restrictions eased, and employment activity could resume. In contrast, a more variable pattern of differences across 2019 and 2020 emerged in men's reporting of job loss, although the significant differences revealed that men reported significantly more job loss in June (6.13%) and August (9.24%) 2020 compared to the corresponding months of the previous year (3.40%, 4.91%).

3.1.2 Resigned from your Job

Table 3 (see main text) shows a stable pattern in the proportion of women and men reporting resigning from their job across the four time periods, including during the months of the pandemic (Mar–Sep 2020), supported by non-significant chi-squares (ps < .05). Figure S1 further shows that there was little difference between years across the months of the pandemic for women and men, although women reported significantly lower rates of resignation in July 2020 (0.18%) compared to 2019 (0.98%) and men showed a significant decrease in April 2020 (0.46%) compared to the prior year (2.99%).

3.2 Retirement

3.2.1 Retired

As shown in Table 3 (see main text), women showed a marked increase in reports of the specific event 'retired' during the months of the pandemic (Mar–Sep 2020; $\chi^2(3) = 10.65$, $p = .001$). Conversely, men showed more variable proportions across time periods that were not significantly different from each other ($\chi^2(3) = 2.05$, $p = .152$). As shown in Figure S1, women reported significantly higher rates of retirement in May 2020 (5.32%) compared to May 2019 (2.37%). In contrast, reported retirement for men was more variable between years, with the only significant increase from 2019 occurring in June 2020 (8.37%).

3.3 Employment Changes

3.3.1 Change of Job

Table 3 (see main text) shows a stable pattern in the proportion of women and men reporting a change of job across the four time periods, supported by non-significant chi-squares (ps < .05). Figure S1 further shows that, although there was a variable pattern of differences, these were not significant across years between Mar–Sep 2020 for women and men.

3.3.2 Reduced/Lost Work Hours

As shown in Table 3 (see main text), the proportion of women reporting reduced/lost work hours increased during the months of the pandemic (Mar–Sep 2020; $\chi^2(3) = 8.47$, $p = .004$), whereas men showed no significant differences ($\chi^2(3) = 0.04$, $p = .836$). Figure S1 shows that, although overall proportions of women reporting reduced/lost work hours increased during the months of the pandemic, the specific differences across years for each month revealed no significant differences from Mar–Sep 2020.

3.3.3 Looking for a Job

Table 3 (see main text) shows a stable pattern in the proportion of women and men reporting the specific event of 'looking for a job' across the four time periods, supported by non-significant chi-

squares (*p*s < .05). Figure S1 further shows that there were no significant differences across years between Mar–Sep 2020 for women and men, except for a significant decrease among women in April 2020 (0.18%) compared to the year before (1.45%).

3.4 Workplace Issues

3.4.1 Stress (Significant)

Table 3 (see main text) shows a stable pattern in the proportion of women and men reporting stress across the four time periods, supported by non-significant chi-squares (*p*s < .05). Figure S1 further illustrates that there were no significant differences across years between Mar–Sep 2020 for women and men, except for a significant decrease among men in April 2020 (0.19%) compared to the year before (1.49%).

3.4.2 Workplace Harassment or Bullying

As shown in Table 3 (see main text), women and men reporting workplace harassment or bullying showed a stable pattern across the four time periods, supported by non-significant chi-squares (*p*s < .05). Figure S1 further demonstrates that there were no significant differences across years for women and men.

3.4.3 Job Insecurity

As shown in Table 3 (see main text), the proportion of women reporting job insecurity increased during the months of the pandemic (Mar–Sep 2020; $\chi^2(3) = 5.67$, $p = .034$), whereas men showed no significant differences ($\chi^2(3) = 0.00$, $p = 1.00$). Figure S1 shows that there were no significant differences across years between Mar–Sep 2020 for women and men, except for a significant increase among women in July 2020 (0.88%) compared to the year before (0.00%).

3.5 Financial Concerns

3.5.1 Decreased Income

As shown in Table 3 (see main text), women and men reporting that their income decreased showed a stable pattern across the four time periods, supported by non-significant chi-squares (*p*s < .05). Figure S1 further shows that there were no significant differences across years between Mar–Sep 2020 for women and men, except for a significant decrease among men in April 2020 (0.19%) compared to the year before (1.49%).

3.5.2 Financial Difficulties

As shown in Table 3 (see main text), women and men reporting financial difficulties showed a stable pattern across the four time periods, supported by non-significant chi-squares (*p*s < .05). Figure S1 further illustrates that there were no significant differences across years between Mar–Sep 2020 for women and men, except for a significant decrease among men in April 2020 (0.00%) compared to the year before (1.49%).

3.6 Summary

We found that the most commonly reported events under each economic event category showed a similar pattern of differences as the broader event categories. For example, involuntary job loss, but not voluntary job loss (i.e., resignation), increased for women and men in the months following

lockdown when normal employment activity resumed with fewer resources. Retirement among women also increased following lockdown. However, other events, including change of job, financial difficulties, job insecurity, and workplace harassment, showed inconsistent differences, but hinted that: (a) stress decreased among men during lockdown, (b) less women were looking for a job in lockdown, (c) job insecurity increased among women following lockdown, and (d) men reported fewer financial difficulties or reduced income during lockdown.

3.7 Figure S1. Percent Occurrence of Most Frequently Reported Specific Economic Events (**A–K**) for Women and Men in Data Collection Wave Prior to Pandemic (Oct 2018–Sep 2019) Vs Wave When Pandemic Occurred (Oct 2019–Sep 2020).

Note. * to the right of the line indicates a significant difference ($p < .01$) between the two time points within that month.
+ to the right of the line indicates a significant difference ($p < .05$) between the two time points within that month.
Grey shading indicates the months of the pandemic occurring in New Zealand.
Standard errors for each proportion across months for the two waves are provided inside each point.

**COVID-19 Timeline**	31 Dec 2019–27 Feb 2020	28 Feb 2020–25 Mar 2020	26 Mar 2020–27 Apr 2020	28 Apr 2020–13 May 2020	14 May 2020–08 Jun 2020	09 Jun 2020–11 Aug 2020	12 Aug 2020–30 Aug 2020	30 Aug 2020–07 Oct 2020
	First cases in Wuhan	First case in NZ	Lockdown – Alert Level 4	Alert Level 3	Alert Level 2	Alert Level 1	Alert Level 3 (Auckland)	Alert Level 2 (Auckland)

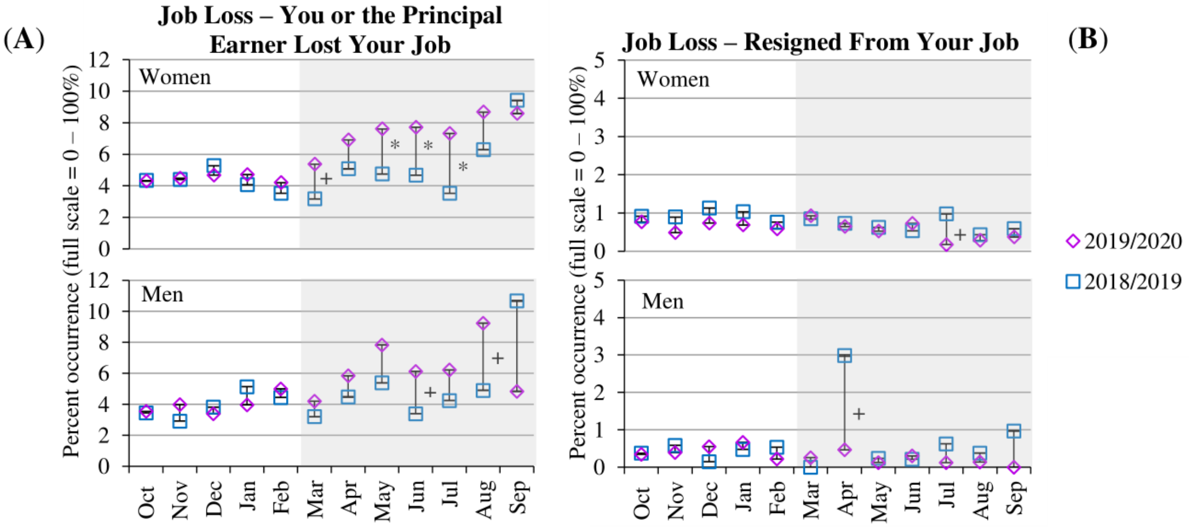

**COVID-19 Timeline**	31 Dec 2019–27 Feb 2020	28 Feb 2020–25 Mar 2020	26 Mar 2020–27 Apr 2020	28 Apr 2020–13 May 2020	14 May 2020–08 Jun 2020	09 Jun 2020–11 Aug 2020	12 Aug 2020–30 Aug 2020	30 Aug 2020–07 Oct 2020
	First cases in Wuhan	First case in NZ	Lockdown – Alert Level 4	Alert Level 3	Alert Level 2	Alert Level 1	Alert Level 3 (Auckland)	Alert Level 2 (Auckland)

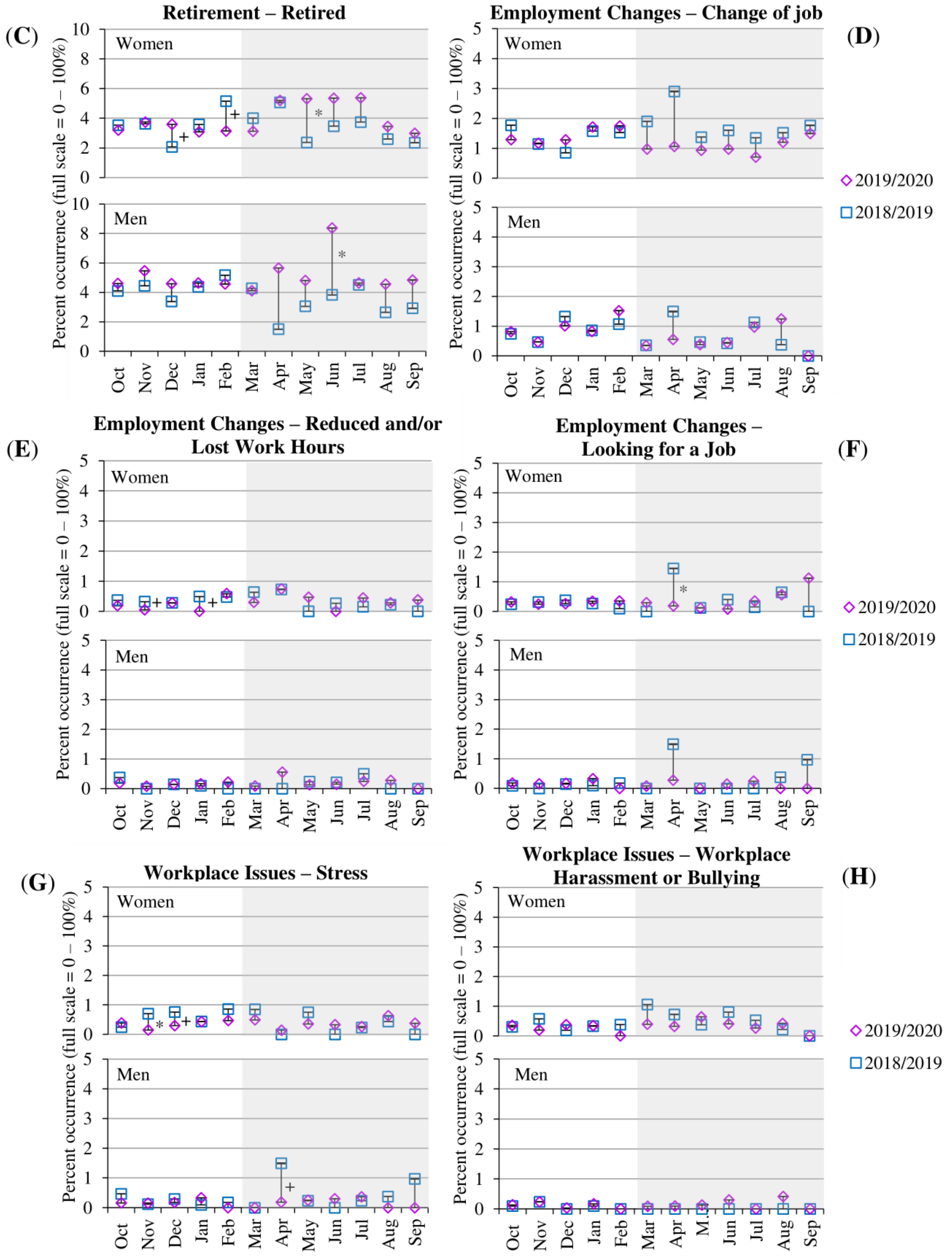

COVID-19 Timeline	31 Dec 2019–27 Feb 2020	28 Feb 2020–25 Mar 2020	26 Mar 2020–27 Apr 2020	28 Apr 2020–13 May 2020	14 May 2020–08 Jun 2020	09 Jun 2020–11 Aug 2020	12 Aug 2020–30 Aug 2020	30 Aug 2020–07 Oct 2020
	First cases in Wuhan	First case in NZ	Lockdown – Alert Level 4	Alert Level 3	Alert Level 2	Alert Level 1	Alert Level 3 (Auckland)	Alert Level 2 (Auckland)

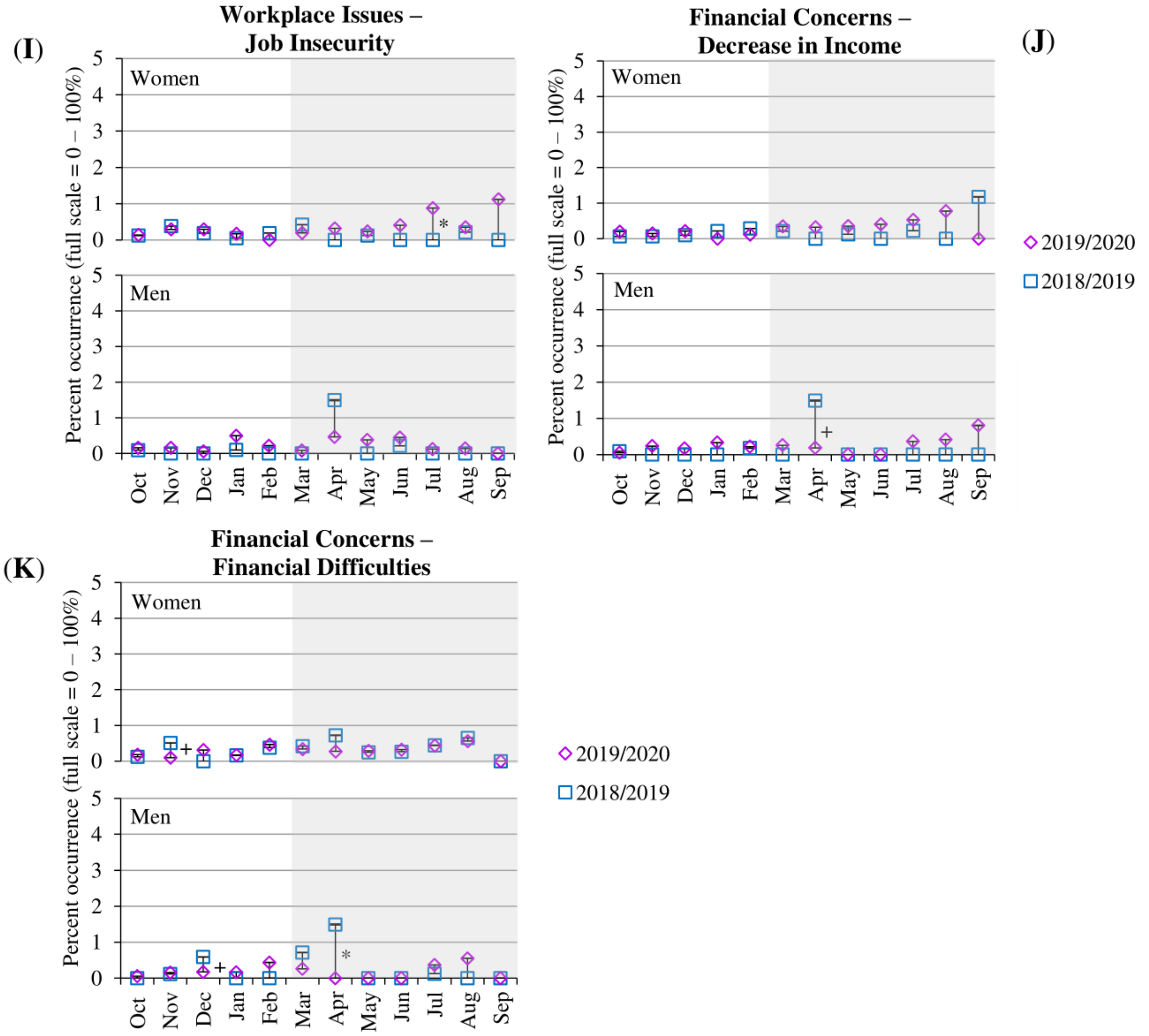

4 Analyses of Top Specific Events under the Social Events Categories

4.1 Relationship Breakdown

4.1.1 Separated from your Spouse/Romantic Partner

Table 4 (see main text) shows a variable pattern in the proportion of women and men reporting a relationship separation across the four time periods, including during the months of the pandemic (Mar–Sep 2020), supported by non-significant chi-squares (*ps* < .05). Figure S2 further shows that there were no significant differences across years between Mar–Sep 2020 for women and men, except for a significant decrease among women in March 2020 (4.15%) compared to the year before

(7.17%) and a significant increase among women in August 2020 (4.23%) compared to August 2019 (2.17%).

4.1.2 Got Divorced

Table 4 (see main text) shows a stable pattern in the proportion of women and men reporting divorce across the four time periods, supported by non-significant chi-squares (*p*s < .05). Figure S2 further demonstrates that, although there was a variable pattern of differences, these were not significant across years between Mar–Sep 2020 for women and men.

4.2 Family Connection

4.2.1 Increased Support to and/or from Family and Friends

As shown in Table 4 (see main text), women and men reporting increased support to or from family and friends showed a stable pattern across the four time periods, supported by non-significant chi-squares (*p*s < .05). Figure S2 further shows that there were no significant differences across years between Mar–Sep 2020 for women and men, except for a significant decrease among men in August 2020 (0.00%) compared to the year before (0.76%).

4.2.2 Increased Time Spent with Family

As shown in Table 3 (see main text), the proportion of women reporting increased time spent with family increased during the months of the pandemic (Mar–Sep 2020; $\chi^2(3) = 4.21$, $p = .040$), whereas men showed no significant differences ($\chi^2(3) = 1.61$, $p = .205$). Figure S2 shows that, although overall proportions of women reporting more time spent with family increased during the months of the pandemic, the specific differences across years for each month revealed no significant differences from Mar–Sep 2020. However, men showed a significant decrease in time spent with family in April 2020 (0.00%) compared to the year before (1.49%).

4.3 Family Troubles

4.3.1 Trouble with Family Members

Table 4 (see main text) shows a stable pattern in the proportion of women and men reporting trouble with family members across the four time periods, supported by non-significant chi-squares (*p*s < .05). Figure S2 further shows no significant differences across years for women and men from March 2020 onwards (when the pandemic occurred in New Zealand).

4.3.2 Isolation from Friends and Family

As shown in Table 4 (see main text), the proportion of women reporting isolation from loved ones increased during the months of the pandemic (Mar–Sep 2020; $\chi^2(3) = 19.28$, $p < .001$), whereas men showed a more stable pattern of proportions across time periods that did not significantly differ ($\chi^2(3) = 0.29$, $p = .590$). As shown in Figure S2, women reported significantly higher rates of isolation from loved ones from March 2020 onwards, but these rates were only significantly higher in May (1.05%), July (1.41%), and August (1.48%) of 2020 compared to the same months in 2019 (0.25%, 0.00%, 0.22%).

4.3.3 Relationship Breakdown for Family Member

Table 4 (see main text) shows a stable pattern in the proportion of women and men reporting a relationship breakdown for a family member across the four time periods, supported by non-significant chi-squares (*p*s < .05). Figure S2 further shows that there were no significant differences across years between Mar–Sep 2020 for women and men, except for a significant decrease among women in May 2020 (0.06%) compared to the year before (0.50%) and a significant decrease among men in August 2020 (0.00%) compared to August 2019 (0.76%).

4.4 Traumatic Interpersonal Events

4.4.1 Someone Assaulted You, Abused You, or Attacked You

Table 4 (see main text) shows a relatively stable pattern in the proportion of women and men reporting the specific event ‘someone assaulted you, abused you, or attacked you’ across the four time periods, supported by non-significant chi-squares (*p*s < .05). Figure S1 further illustrates no significant differences across years for women and men from March 2020 onwards.

4.4.2 Someone Sexually Harassed You

Table 4 (see main text) shows a significant increase in the proportion of women reporting sexual harassment during the months of the pandemic (Mar–Sep 2020; $\chi^2(3) = 7.44$, $p = .006$), whereas men showed no significant differences ($\chi^2(3) = 0.00$, $p = .960$). As shown in Figure S2, women reported higher rates of sexual harassment between Mar–Sep 2020, but only May 2020 (2.75%) showed a significant increase compared to the same month in 2019 (0.75%).

4.5 Summary

We found that many of the most frequently reported events under the social event categories showed inconsistent differences, but hinted that (a) separation decreased among women during lockdown but increased during the second regional outbreak, (b) men reported less time spent with family during the national lockdown and less support from family and friends during the second regional outbreak. We also found that isolation from loved ones was more salient or experienced for women during the months following lockdown when restrictions eased, perhaps because reconnecting loved ones was more difficult than expected or the sense of connection felt while being at home was lost when normal activities resumed. We also found that women reported more sexual harassment following national lockdown when people were confined to their homes for an extended time period.

4.6 **Figure S2.** Percent Occurrence of Most Frequently Reported Specific Social Events (**A–I)** for Women and Men in Data Collection Wave Prior to Pandemic (Oct 2018–Sep 2019) Vs Wave When Pandemic Occurred (Oct 2019–Sep 2020).

Note. * to the right of the line indicates a significant difference ($p < .01$) between the two time points within that month.
+ to the right of the line indicates a significant difference ($p < .05$) between the two time points within that month.
Grey shading indicates the months of the pandemic occurring in New Zealand.
Standard errors for each proportion across months for the two waves are provided inside each point.

**COVID-19 Timeline**	31 Dec 2019–27 Feb 2020	28 Feb 2020–25 Mar 2020	26 Mar 2020–27 Apr 2020	28 Apr 2020–13 May 2020	14 May 2020–08 Jun 2020	09 Jun 2020–11 Aug 2020	12 Aug 2020–30 Aug 2020	30 Aug 2020–07 Oct 2020
	First cases in Wuhan	First case in NZ	Lockdown – Alert Level 4	Alert Level 3	Alert Level 2	Alert Level 1	Alert Level 3 (Auckland)	Alert Level 2 (Auckland)

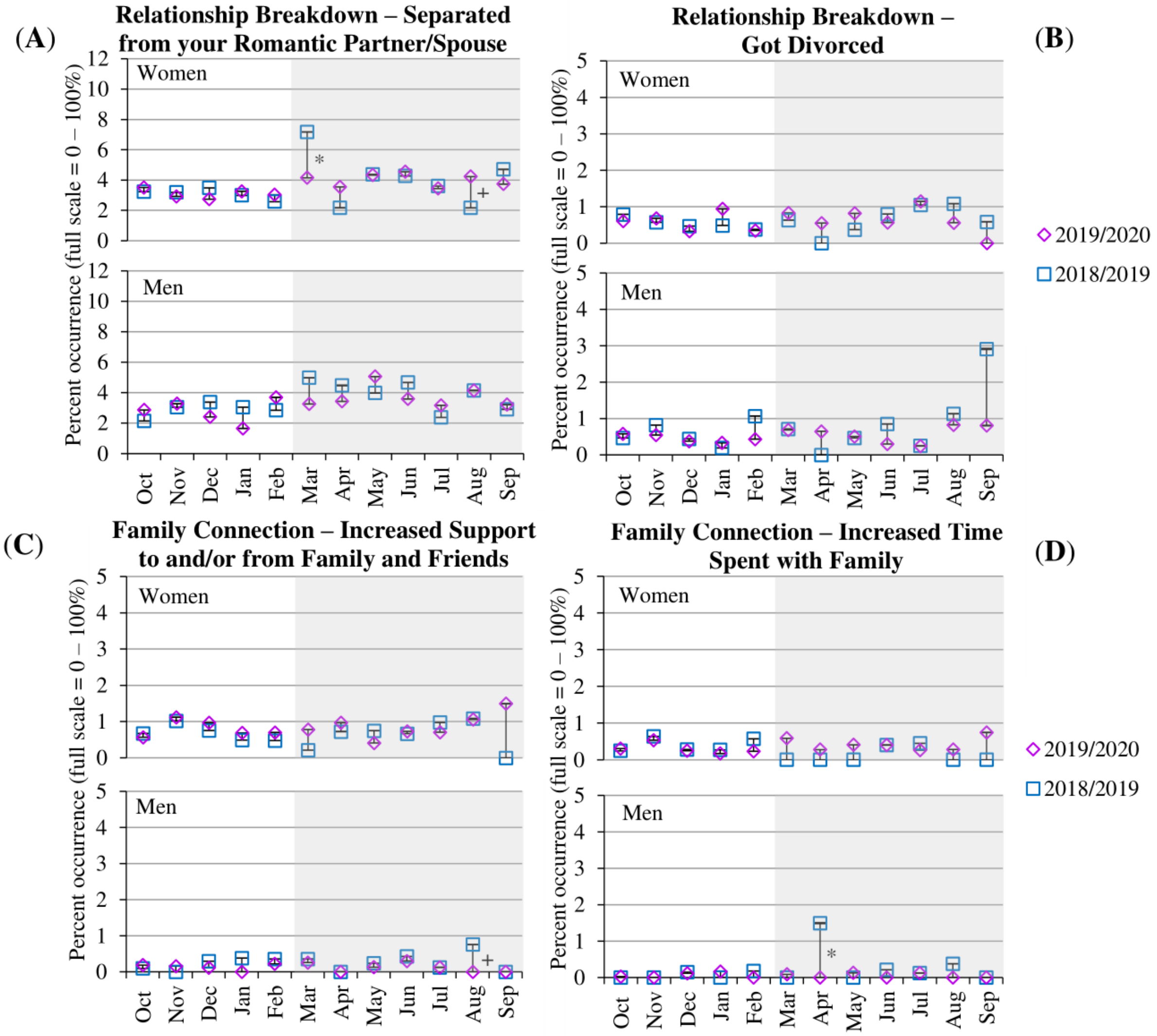

COVID-19 Timeline	31 Dec 2019–27 Feb 2020	28 Feb 2020–25 Mar 2020	26 Mar 2020–27 Apr 2020	28 Apr 2020–13 May 2020	14 May 2020–08 Jun 2020	09 Jun 2020–11 Aug 2020	12 Aug 2020–30 Aug 2020	30 Aug 2020–07 Oct 2020
	First cases in Wuhan	First case in NZ	Lockdown – Alert Level 4	Alert Level 3	Alert Level 2	Alert Level 1	Alert Level 3 (Auckland)	Alert Level 2 (Auckland)

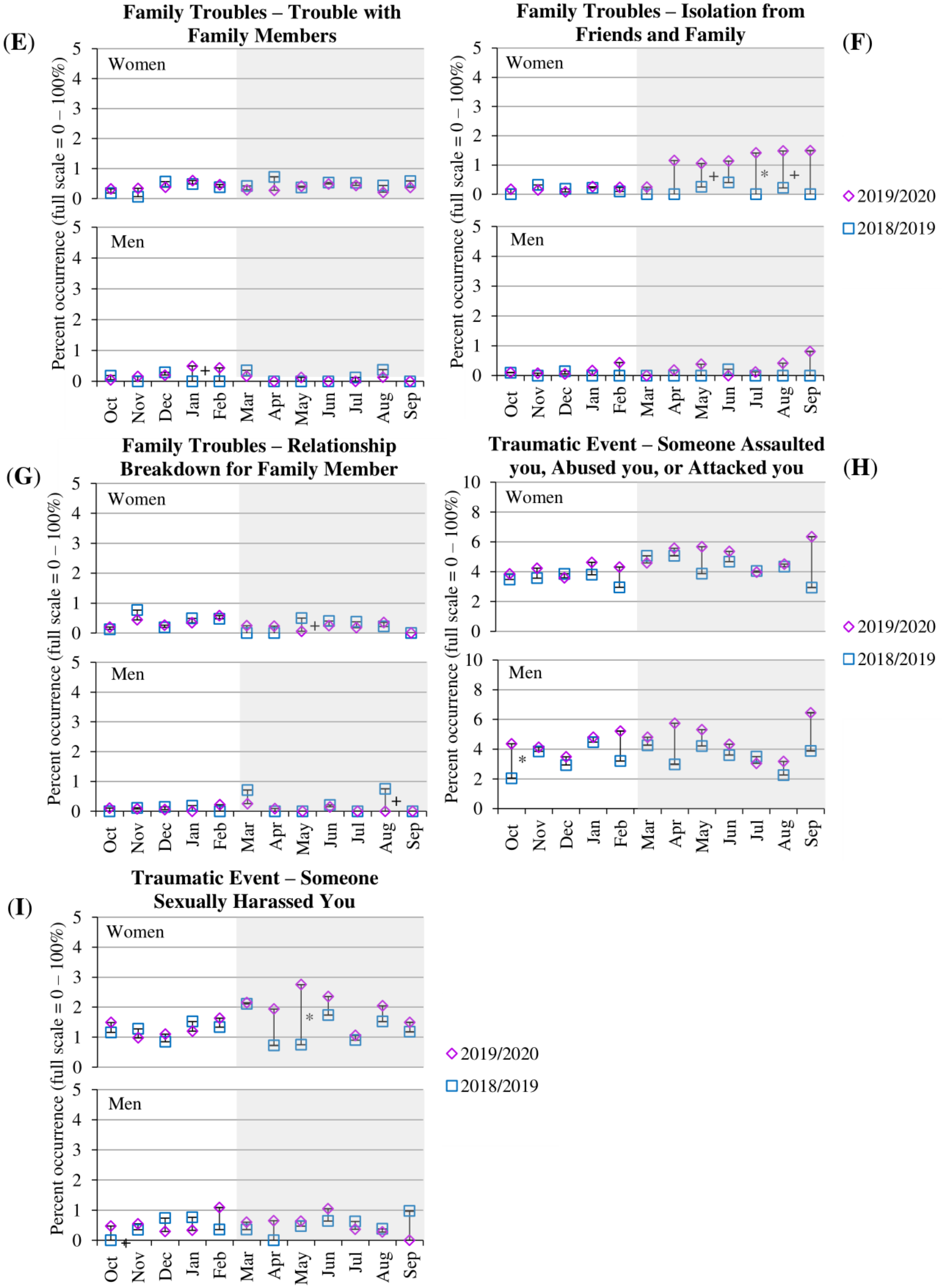

5 Analyses of Top Specific Events under the Well-Being Events Categories

5.1 Negative Lifestyle Changes

5.1.1 Less Social Activities or Recreation

As shown in Table 5 (see main text), women and men reporting less participation in social activities or recreation increased in the during the months of the pandemic (Mar–Sep 2020) compared to the other three time periods (women ($\chi^2(3) = 78.38$, $p < .001$; men ($\chi^2(3) = 6.81$, $p = .009$). As shown in Figure S3, women began reporting fewer social or recreational activities in March 2020 when the pandemic emerged in New Zealand and continued through to June, ranging from 1.22% to 8.34%, all of which showed a significant increase from 2019. One other significant increase was observed in August 2020 (2.19%), compared to the same month in 2019 (0.00%). In contrast, men showed a more variable pattern, with only one significant increase May 2020 (3.67%) compared to the previous year (0.47%).

5.2 Mental Health

5.2.1 Mental Health Problem of a Family Member

Table 5 (see main text) shows a stable pattern in the proportion of women and men reporting a mental health problem of a family member across the four time periods, including during the months of the pandemic (Mar–Sep 2020), supported by non-significant chi-squares (ps < .05). Figure S3 further demonstrates no significant differences across years for women and men.

5.2.2 Depression

Table 5 (see main text) shows a stable pattern in the proportion of women and men reporting depression across the four time periods, supported by non-significant chi-squares (ps < .05). Figure S3 further shows no significant differences across years for women and men from March 2020 onwards.

5.2.3 Anxiety

Table 5 (see main text) shows a stable pattern in the proportion of women and men reporting anxiety across the four time periods, supported by non-significant chi-squares (ps < .05). Figure S3 further illustrates no significant differences across years for women and men from March 2020 onwards, except for a significant decrease in anxiety among men in April 2020 (0.09%) compared to April 2019 (1.49%).

5.3 Summary

We found that the most frequently reported events under each well-being category showed a similar pattern of differences as the broader event categories. In particular, we found that less social activities and recreation was an event that was persistently more salient or experienced for women during the months of the pandemic. However, the specific mental health events of ‘mental health problem of family member’, ‘depression’, and ‘anxiety’ remained relatively unchanged pre- versus post-pandemic, suggesting that many people were resilient throughout the pandemic.

5.4 Figure S3. Percent Occurrence of Most Frequently Reported Specific Well-Being Events (**A, B, C, D)** for Women and Men in Data Collection Wave Prior to Pandemic (Oct 2018–Sep 2019) Vs Wave When Pandemic Occurred (Oct 2019–Sep 2020).

Note. * to the right of the line indicates a significant difference ($p < .01$) between the two time points within that month.
+ to the right of the line indicates a significant difference ($p < .05$) between the two time points within that month.
Grey shading indicates the months of the pandemic occurring in New Zealand.
Standard errors for each proportion across months for the two waves are provided inside each point.

**COVID-19 Timeline**	31 Dec 2019–27 Feb 2020	28 Feb 2020–25 Mar 2020	26 Mar 2020–27 Apr 2020	28 Apr 2020–13 May 2020	14 May 2020–08 Jun 2020	09 Jun 2020–11 Aug 2020	12 Aug 2020–30 Aug 2020	30 Aug 2020–07 Oct 2020
	First cases in Wuhan	First case in NZ	Lockdown – Alert Level 4	Alert Level 3	Alert Level 2	Alert Level 1	Alert Level 3 (Auckland)	Alert Level 2 (Auckland)

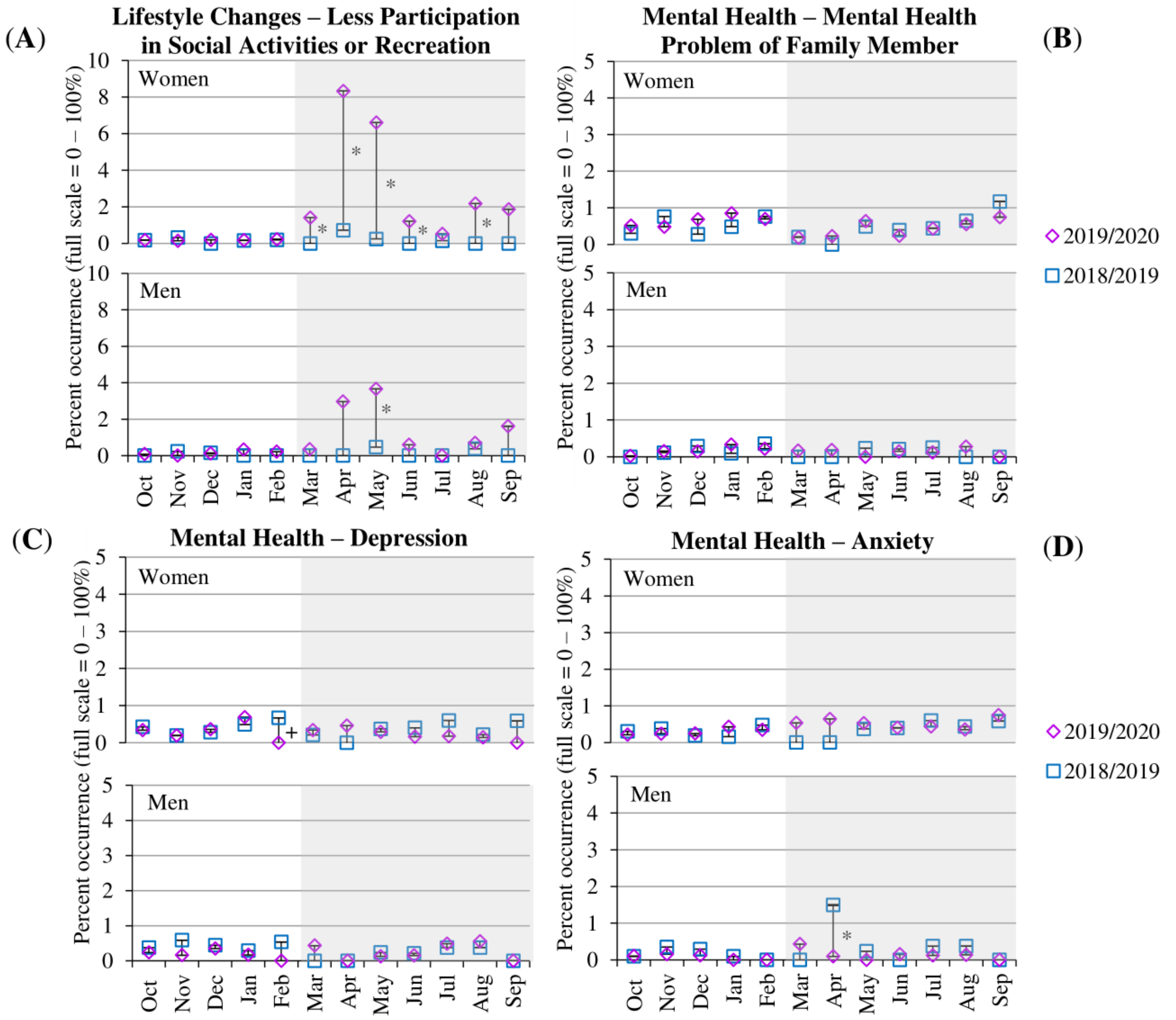

Supplement: Supplementary file 1 [file Data_Sheet_1.pdf]
